# Amodiaquine Modulates Aggregation and Disassembly of Amyloid-β and Tau and Attenuates Neuroinflammatory Responses and Aβ Production

**DOI:** 10.3390/pharmaceutics17111417

**Published:** 2025-10-31

**Authors:** Sinae Jang, Sujin Kim, Na-Hyun Kim, Soo Jung Shin, Vijay Kumar, Jeong Gyu Son, Minseok Lee, Choon-gil Kim, Eun-Kyung Lim, Hyunju Chung, Young Ho Koh, Yunkwon Nam, Minho Moon

**Affiliations:** 1 Department of Biochemistry, College of Medicine, Konyang University, 158, Gwanjeodong-ro, Seo-gu, Daejeon 35365, Republic of Korea; syjjang4478@naver.com (S.J.); kxujin@korea.kr (S.K.); uss02036@naver.com (N.-H.K.); tlstnzz83@gmail.com (S.J.S.); vijay10187@gmail.com (V.K.); son@konyang.ac.kr (J.G.S.); dlalstjr1322@naver.com (M.L.); 2 Division of Cardiovascular Disease Research, Department of Chronic Disease Convergence Research, Korea National Institute of Health, 187 Osongsaengmyeong2(i)-ro, Osong-eup, Heungdeok-gu, Cheongju-si 28159, Republic of Korea; 3 Flowmedi, 160 Techno 2-ro, Yuseong-gu, Daejeon 34028, Republic of Korea; joykim@flowmedi.co.kr; 4 Bionanotechnology Research Center, Korea Research Institute of Bioscience and Biotechnology (KRIBB), 125 Gwahak-ro, Yuseong-gu, Daejeon 34141, Republic of Korea; eklim1112@kribb.re.kr; 5 Department of Nanobiotechnology, KRIBB School of Biotechnology, University of Science and Technology, 217 Gajeong-ro, Yuseong-gu, Daejeon 34113, Republic of Korea; 6 Department of Core Research Laboratory, Medical Science Research Institute, Kyung Hee University Hospital Gangdong, Seoul 05278, Republic of Korea; hjchung@khnmc.or.kr; 7 Division of Brain Diseases Research, Department of Chronic Disease Convergence Research, Korea National Institute of Health, 187 Osongsaengmyeong2(i)-ro, Osong-eup, Heungdeok-gu, Cheongju-si 28159, Republic of Korea; kohyoungho122@gmail.com; 8 Research Institute for Dementia Science, Konyang University, 158, Gwanjeodong-ro, Seo-gu, Daejeon 35365, Republic of Korea

**Keywords:** Alzheimer’s disease, amyloid beta, tau, aggregation inhibitor, amodiaquine

## Abstract

**Background**: Alzheimer’s disease (AD) is a progressive neurodegenerative disorder characterized by the accumulation of amyloid-β (Aβ) plaques and hyperphosphorylated tau tangles, which synergistically accelerate disease progression. Since Aβ plaques and tau tangles are key factors in the development of AD, dual-targeting of Aβ and tau aggregation represents a promising therapeutic strategy. Amodiaquine (AQ), a quinoline-based antimalarial, has recently attracted attention for its ability to suppress protein aggregation. However, direct effects of AQ on both Aβ and tau aggregation remain unclear. **Methods**: The effects of AQ on the aggregation and dissociation of Aβ and tau were examined using a thioflavin T (ThT) assays. Molecular docking and molecular dynamics (MD) simulations were performed to examine binding characteristics and structural interactions. The effects of AQ on the expression of pro-inflammatory cytokines induced by Aβ and tau aggregation in BV2 microglial cells were analyzed by qRT-PCR. **Results**: ThT assay demonstrated a dose-dependent dual effect of AQ on Aβ, where 25 μM inhibited aggregation after 36 h, while 250 μM markedly accelerated it, reaching a plateau within 12 h. All concentrations of AQ promoted the disassembly of mature Aβ fibrils within 12 h. Molecular docking revealed stronger binding of AQ to aggregated Aβ (−45.17 and −23.32 kcal/mol for pentameric 2BEG and hexameric 2NAO) than to monomeric Aβ (−4.81 and −7.29 kcal/mol for 1Z0Q and 2BEG). MD simulation suggested that AQ disrupted the cross-β-sheet interactions of Aβ aggregates. In the case of tau, ThT assay showed that all concentrations of AQ inhibited tau aggregation from 6 h, and 350 μM AQ promoted the disassembly of mature fibrils from 6 h. Molecular docking indicated stronger binding of AQ to aggregated tau (−27.95 and −12.13 kcal/mol for the pentameric and decameric 5O3L) than to monomeric tau (−3.05 kcal/mol for 8Q96). MD simulations revealed no major structural changes in the aggregates. In BV2 cells, 1 and 10 μM AQ significantly reduced Aβ and tau-induced TNF-α and IL-6 mRNA expressions. In APP-H4 cells, 10 μM AQ decreased the level of Aβ compared to the control. **Conclusions**: AQ modulates both Aβ and tau aggregation and attenuates neuroinflammation and reduces Aβ pathology, supporting its potential as a dual-target therapeutic candidate for AD.

## 1. Introduction

Alzheimer’s disease (AD) is a complex neurodegenerative disorder caused by a combination of multiple factors. Since the pathogenesis of AD is highly intricate, various neuropathological features have been observed in the brain with AD, leading to the development of diverse hypotheses, including the amyloid-β (Aβ) cascade, tau, and inflammation hypotheses [1]. However, the presence of Aβ-containing extracellular plaques and hyperphosphorylated tau-containing intracellular neurofibrillary tangles (NFTs) in the brain with AD remains an indisputable hallmark [2], underscoring the rationale for targeting Aβ and tau in therapeutic strategies. Aggregation and accumulation of Aβ and hyperphosphorylated tau occur approximately 20 years before the onset of AD [3,4]. Interestingly, during the aggregation process, both Aβ and hyperphosphorylated tau undergo a conformational change into β-sheet-rich structures [5]. Aggregates of Aβ and hyperphosphorylated tau, which are stacked in a β-sheet structure, cause neuroinflammation as well as neurotoxicity and are closely associated with the pathogenesis and progression of AD [6]. Moreover, Aβ and tau accelerate AD-related pathological changes, such as neuroinflammation and neurodegeneration, through synergistic effects that affect each other [7,8]. Therefore, since the aggregation of both Aβ and tau plays important roles in various aspects of AD pathology, effectively modulating these disordered proteins in AD represents a promising strategy for the treatment of AD [9].

Amodiaquine (AQ) is an antimalarial agent based on a quinoline scaffold, which serves as a core structural framework for its pharmacological activity [10]. Notably, many studies have reported that quinoline derivatives and quinoline-based hybrids inhibit the aggregation of Aβ and tau [11,12,13,14]. Quinoline derivatives prevent the formation of tau aggregates by binding to the C-terminus of tau involved in the formation of paired helical filaments (PHFs) [13]. The steroid–quinoline hybrid not only inhibited the aggregation of Aβ but also promoted the dissociation of aggregated Aβ [11]. Additionally, some quinolinone hybrids suppressed Aβ aggregation and acetylcholinesterase activity [14]. Interestingly, AQ has been shown to have potential in novel approaches, such as inhibition of cholinesterase, in addition to its antimalarial activity [15]. Moreover, AQ blocked the fibrillation of human lysozyme by inhibiting aggregation and promoting dissociation of aggregates in human lysozyme amyloidosis was typically observed [16]. This finding suggests that AQ could serve as a potential therapeutic agent for amyloidopathies, including AD. Indeed, our previous studies demonstrated that AQ alleviated various AD-related pathologies, including Aβ pathology, in a 5XFAD mouse model [17]. In addition, we have previously confirmed that AQ treatment modulates the expression of key cell cycle–related regulators like E2F transcription factor 1 (E2F1), cyclin A, and cyclin-dependent kinase 2 (CDK2) in adult rat hippocampal neural stem cells [18]. However, the mechanisms by which AQ ameliorates AD-related pathology, including the direct effects on Aβ and tau aggregation and disassembly, and the associated molecular pathways, have not been fully elucidated.

In this study, we investigated the effect of AQ on the aggregation of Aβ and tau and the dissociation of their fibrils using a thioflavin T (ThT) assay. To further explore the underlying mechanisms by which AQ modulates the aggregation of Aβ and tau, we performed molecular docking simulations to predict the binding characteristics of AQ with various conformations of Aβ and tau. In addition, molecular dynamics (MD) simulations were conducted to track structural changes in AQ–Aβ and AQ–tau complexes over simulation time, providing insights into their intermolecular interactions. Moreover, we assessed the effects of AQ on the secretion of Aβ and tau-induced pro-inflammatory cytokines in BV2 microglial cells through the qRT-PCR analysis. Finally, we estimated the pharmacokinetic properties of AQ using the web-based software PreADMET version 2.0. Taken together, the present study aimed to elucidate the molecular mechanisms through which AQ modulates Aβ and tau aggregation and disassembly, and to evaluate the therapeutic potential of AQ as a dual-target modulator for AD.

## 2. Materials and Methods

### 2.1. Evaluation of the Anti-Aggregation and Disaggregation Effects of AQ on Aβ_42_

Aβ_42_ peptides (Product number: 4014447; Bachem AG, Bubendorf, Switzerland) were commercially purchased and were dissolved in 0.1 M aqueous ammonia to prevent self-aggregation [19]. To evaluate the anti-aggregation effect of AQ on Aβ, a ThT fluorescence assay was performed using non-aggregated Aβ_42_ incubated with or without AQ. AQ (Sigma-Aldrich, St. Louis, MO, USA) was dissolved in triple distilled water and ThT (Tokyo Chemical Industry Co., Ltd., Tokyo, Japan) was prepared in 50 mM glycine buffer (pH 8.9). Aβ_42_ peptides in aqueous ammonia were diluted with triple distilled water to a final concentration of 25 μM and transferred into black polypropylene 96-well plates (SPL Life Science, Pocheon-si, Republic of Korea). Aβ_42_ dissolved in triple distilled water undergoes spontaneous self-aggregation. AQ was added to the wells at concentrations of 2.5, 25, and 250 μM, along with 100 μM morin (positive control) and 15 μM ThT solution. The mixtures were incubated at 37 °C for 48 h. Next, to assess the disaggregation effect of AQ on β-sheet-rich Aβ_42_ aggregates, preformed Aβ_42_ fibrils were prepared by incubating 25 μM Aβ_42_ for 48 h, the time point at which the ThT fluorescence intensity reached its maximum (Figure S1A). The samples were then treated with 2.5, 25, or 250 μM of AQ and 15 μM of ThT solution and incubated at 37 °C for 48 h. The fluorescence intensity was measured using a SpectraMax iD3 multi-mode microplate reader (Molecular Devices, San Jose, CA, USA) with excitation and emission wavelengths set to 440 nm and 484 nm, respectively. No spectral interference was observed between ThT and three concentrations of AQ (Figure S1B).

### 2.2. Molecular Docking Simulation of AQ with Aβ or Tau

Molecular docking simulations were conducted using BIOVIA Discovery Studio Client 2024 (Biovia, San Diego, CA, USA). The 3D structures of AQ (PubChem CID: 2165), morin (PubChem CID: 5281670), and methylene blue (PubChem CID: 6099) were retrieved from the PubChem database. Various Aβ and tau protein structures relevant to aggregation and pathogenic conformations were selected from the Protein Data Bank (Table 1). Docking was carried out using the standard protocol in Discovery Studio, which employs the Chemistry at HARvard Macromolecular Mechanics (CHARMm) force field for semi-rigid docking of small molecules. Proteins and ligands were preprocessed with appropriate structural modifications, and binding sites were defined based on our previous study [20]. To improve the accuracy of docking results, the number of top poses was set to 10, and the pose cluster radius was adjusted to 0.5 Å. Protein–ligand interactions were visualized in both 2D and 3D using visualization tools in Discovery Studio Client 2024.

### 2.3. MD Simulations of AQ with Aβ or Tau

MD simulations were executed using BIOVIA Discovery Studio Client 2024. The top-ranked docking poses of AQ bound to Aβ or tau, identified from molecular docking results, were used as input structures for the MD simulations. Specifically, the pentameric forms of Aβ (PDB ID: 2BEG) and tau (PDB ID: 5O3L), both in the presence and absence of AQ, were selected for analysis. MD simulations were carried out using the CHARMm force field, in which all interatomic interactions are defined by CHARMm parameters. The structures were solvated in a virtual water box, and the standard dynamics cascade protocol was followed. This protocol consists of the following steps: Minimization 1, Minimization 2, Heating, Equilibration, Production, and Advanced dynamics. Minimization 1 was designed using the steepest descent algorithm with 1000 maximum steps and a root mean square (RMS) gradient of 1.0. Minimization 2 was carried out using the adopted basis Newton-Raphson (NR) algorithm with a maximum of 2000 steps and an RMS gradient of 0.1. Next, the Heating step lasted for 50 picoseconds (ps), with the system temperature gradually increased from 50 K to 300 K. Subsequently, the Equilibration step was conducted for 100 ps at 300 K. Finally, the Production and advanced steps were performed for 4000 ps, using a Leapfrog Verlet integrator. A nonbonded interaction cutoff of 10.0 Å (lower) and 12.0 Å (upper) was applied, and trajectory frames were saved every 20 ps. MD studies were conducted using the NVE (microcanonical) and NPT (isothermal-isobaric) ensembles. In the NVE ensemble, the number of particles (N), system volume (V), and total energy (E) were conserved throughout the simulation. This ensemble is thus suitable for monitoring intrinsic molecular dynamics, verifying energy conservation, and evaluating short-term structural stability. In the NPT ensemble, the number of particles (N), pressure (P), and temperature (T) were conserved throughout the simulation, allowing energy (and volume) exchange with a reservoir. The NPT ensemble better mimics real-world conditions where systems exchange energy and/or volume with their environment.

### 2.4. Evaluation of the Anti-Aggregation and Disaggregation Effects of AQ on Tau K18

Recombinant tau K18 fragments (Keyprogen, Daejeon, Republic of Korea) were commercially purchased [20,21]. The purity of tau K18 was analyzed using 20% sodium dodecyl sulfate-polyacrylamide gel electrophoresis (Figure S2). To investigate the anti-aggregation effect of AQ on tau, ThT assays were performed using non-aggregated tau K18 incubated in the presence and absence of AQ. The tau K18 stock was prepared at a concentration of 35 μM in Dulbecco’s phosphate-buffered saline (DPBS; pH 7.4). To induce tau K18 aggregation, 1 mL of a solution containing 0.1 mg/mL heparin (Sigma-Aldrich) and DPBS was added. Heparin was used to promote aggregation of tau K18 fibrillization. Tau K18 and the aggregation-inducing solution were added to black polypropylene 96-well plates, followed by 3.5, 35, and 350 μM of AQ, 100 μM of methylene blue (MB; positive control), and 15 μM of ThT solution. The mixtures were incubated at 37 °C for 24 h. Next, to assess the ability of AQ to disaggregate β-sheet-rich tau K18 aggregates, ThT assays were conducted using preformed tau K18 fibrils incubated in the presence and absence of AQ. Since tau K18 aggregation reached its maximum ThT fluorescence intensity at 24 h (Figure S3A). Subsequently, 3.5, 35, and 350 μM of AQ and 15 μM of ThT solution were added to each well, and the mixtures were incubated at 37 °C for an additional 24 h. Fluorescence intensity was measured using a SpectraMax iD3 multi-mode microplate reader (Molecular Devices) with excitation and emission wavelengths set to 440 nm and 484 nm, respectively. No spectral interference was observed between ThT and three treated concentrations of AQ (Figure S3B).

### 2.5. Cell Culture Conditions of BV2 Cells and APP-H4 Cells

HT22 cells, from murine hippocampal neuronal cell lines, were cultured in Dulbecco’s Modified Eagle’s Medium (DMEM; WelGENE, Gyeongsan-si, Republic of Korea). BV2 cells, mouse microglial cell line, were cultivated in Dulbecco’s Modified Eagle’s Medium/F12 Nutrient Mixture Ham (DMEM/F12; WelGENE), Amyloid precursor protein (APP)-H4 cells (H4 cells overexpressing human APP and producing high levels of Aβ) were maintained in Dulbecco’s Modified Eagles Media (DMEM; WelGENE). HT22 cells, BV2 cells and APP-H4 cells were complemented with 10% fetal bovine serum (WelGENE) and 100 units/mL penicillin-streptomycin (Gibco, Thermo Fisher Scientific, Waltham, MA, USA) and incubated in a humidified 5% CO_2_ incubator at 37 °C.

### 2.6. Cell Viability Assay in HT22 Cells, BV2 Cells and APP-H4 Cells

HT22, BV2 and APP-H4 cells were cultured in a 96-well microplate at a density of 5 × 10^3^ cells/well and incubated at 37 °C for 24 h. To investigate the effect of AQ on neurotoxicity induced by monomeric Aβ_42_, HT22 cells were treated with AQ at doses of 0.001, 0.01, 0.1, 1, and 10 μM for 24 h. BV2 and APP-H4 cells were treated with AQ at doses of 0.01, 0.1, 1, 2, 4, 8, and 10 μM for 24 h. Control groups were treated with the same volumes of medium. After the incubation, 10 μL of water-soluble tetrazolium-1 (WST-1) solution (DoGenBio, Seoul, Republic of Korea) was treated to each well. Before measurement, the cells were shaken for 10 s. Absorbance was detected at 450 nm with a SpectraMax iD3 multi-mode microplate reader (Molecular Devices, USA).

### 2.7. Quantitative Reverse-Transcription Polymerase Chain Reaction (qRT-PCR) Analysis of Inflammatory Cytokines in BV2 Cells

BV2 cells were stimulated with 25 μM Aβ_42_ and treated with AQ for 24 h, or with 10 μM tau K18 and treated with AQ for 6 h. Total RNA was extracted from BV2 cells using the AccuPrep RNA Extraction Kit (Bioneer, Daejeon, Republic of Korea) according to the manufacturer’s instructions, and reverse-transcribed using TaKaRa PrimeScript RT Master Mix (Takara, Shiga, Japan), and the RNA was then synthesized into cDNA. To measure the expression of pro-inflammatory cytokines, qRT-PCR was performed using iQ™ SYBR^®^ Green Supermix (Bio-Rad, Hercules, CA, USA). To validate the expression of cytokines in BV2 cells, the obtained cDNA was amplified with primers specific for Tumor Necrosis Factor-alpha (TNF-α) were as follows: sense, 5′-TCG TAG CAA ACC ACC AAG TG-3′ and antisense, 5′-ATA TAG CAA ATC GGC TGA CG-3′, Interleukin-6 (IL-6) were as follows: sense, 5′-GAG GAT ACC ACT CCC AAC AGA CC-3′ and antisense, 5′-AAG TGC ATC ATC GTT GTT CAT ACA-3′ and glyceraldehyde-3-phosphate dehydrogenase (GAPDH) were as follows: sense, 5′-TGG CAC AGT CAA GGC TGA GA-3′ and antisense, 5′-CTT CTG AGT GGC AGT GAT GG-3′. The cycling conditions comprised 95 °C for 3 min, 40 cycles of 95 °C for 15 s, 59 °C for 30 s, 72 °C for 30 s, and 72 °C for 10 min. The mRNA levels of TNF-α and IL-6 were calculated relative to the amounts of GAPDH and normalized to each individual mRNA. Quantification cycle (Cq) values were normalized to GAPDH Cq. Comparative CT method (2^−ΔΔCq^) was used for analysis. All reactions were conducted using the CFX duet Real-Time PCR Detection System and analyzed using CFX Maestro software version 2.3 (Bio-Rad).

### 2.8. Enzyme-Linked Immunosorbent Assay (ELISA) in APP-H4 Cells

To examine the effects of AQ on Aβ production, the APP-H4 cells were seeded into a 12-well plate at a density of 1 × 10^5^ cells/well. After 24 h, the culture medium was replaced with serum-free media for 1 h. The APP-H4 cells were then treated with 1 or 10 μM of AQ for 24 h. The conditioned media were collected and measured human Aβ_40_ using an ELISA kit (catalog number KHB3481, Thermo Fisher Scientific, Waltham, MA, USA) according to the manufacturer’s instructions. The absorbance of the samples was measured at 450 nm using a SpectraMax iD3 multi-mode microplate reader (Molecular Devices, USA).

### 2.9. Prediction of Pharmacokinetic Characterization

To evaluate the drug-like properties of AQ, a comprehensive analysis of absorption, distribution, metabolism, and excretion (ADME) was performed using web-based computational tools. Physicochemical and pharmacokinetic parameters, including plasma protein binding (PPB), Caco-2 permeability, human intestinal absorption (HIA), blood–brain barrier (BBB) penetration, and inhibition of cytochrome P450 enzymes (CYP 2C19 and CYP 2D6), were predicted using PreADMET (https://preadmet.qsarhub.com; BMDRC, Incheon, Republic of Korea). Additionally, the Log Po/w and total clearance of AQ were predicted using pkCSM (https://biosig.lab.uq.edu.au/pkcsm, accessed on 2 October 2024; University of Queensland, Australia).

### 2.10. Statistical Analysis

All analyses were performed in a randomized, blinded fashion. Statistical analyses were performed using GraphPad Prism10.0 software (GraphPad Software, Inc., San diego, CA, USA). All statistical data are displayed as the mean ± standard deviations (SD). For comparisons between the groups, one-way analysis of variance (ANOVA) and two-way ANOVA followed by Fisher’s least significant difference (LSD) test was used to assess the normality of the data. A *p*-value < 0.05 indicated statistical significance. All error bars represent the means with standard deviations.

## 3. Results

### 3.1. Modulatory Effects of AQ on Aβ_42_

**Figure 1:**
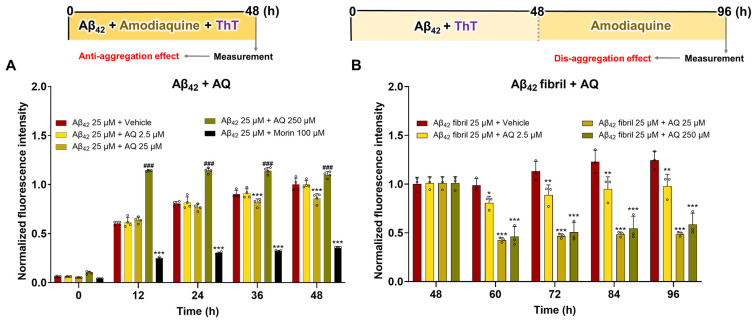
Modulatory effects of AQ on anti- and dis-aggregation of Aβ_42_. A ThT assay to assess the effect of AQ on the (**A**) aggregation of Aβ_42_ and (**B**) dissociation of Aβ fibrils. The vehicle group was treated with triple distilled water. Morin was used as a positive control for Aβ aggregation inhibitor. Values are expressed as means ± standard deviations, n = 3–4 per group. Statistical significance was performed using two-way ANOVA, followed by Fisher’s LSD test. The F-statistics (F) are for (**A**) the row factor (F(4, 75) = 2063, *p* < 0.0001) and column factor (F(4, 75) = 1128, *p* < 0.0001), with a significant interaction detected (F(16, 75) = 77.53, *p* < 0.0001), and (**B**) the row factor (F(4, 40) = 24.68, *p* < 0.0001) and column factor (F(3, 40) = 131.3, *p* < 0.0001), with a significant interaction also detected (F(12, 40) = 8.947, *p* < 0.0001). * *p*-value < 0.05, ** *p*-value < 0.01 and *** *p*-value < 0.001 indicate significant decreases compared with the vehicle-treated Aβ_42_ group, reflecting anti-aggregation or disaggregation effects. ^###^ *p*-value < 0.001 indicate significant increases compared with the vehicle-treated Aβ_42_ group, reflecting pro-aggregation effects.

AQ has been reported to inhibit the fibrillation of human lysozyme, a protein known to form amyloid-like aggregates [16]. However, there are no studies on whether AQ modulates Aβ aggregation. Thus, we performed a ThT assay to evaluate whether AQ inhibits the aggregation of Aβ or promotes the dissociation of Aβ fibrils. To assess the anti-aggregation effect of AQ on Aβ_42_, we treated non-aggregated Aβ_42_ with 2.5 μM, 25 μM, and 250 μM concentrations of AQ and morin as a positive control (Figure 1A). Fluorescence intensity, which reflects Aβ aggregation, was measured over incubation time. Treatment with 2.5 μM AQ showed no difference in fluorescence intensity compared to the vehicle-treated Aβ_42_. Treatment with 25 μM AQ resulted in a significant reduction in fluorescence intensity at the time of administration compared to the vehicle-treated Aβ_42_. This difference was not observed at 12 and 24 h but became significant again at 36 and 48 h, suggesting that AQ may partially exert its effects through transient interactions with specific forms of Aβ_42_. Unexpectedly, treatment with 250 μM AQ showed a higher fluorescence intensity than the vehicle group throughout the entire period from the time of treatment to 48 h, suggesting that a high concentration of AQ may accelerate the aggregation of Aβ_42_. Morin significantly reduced fluorescence intensity compared to the vehicle group at all measured time points. Next, to investigate the disaggregation effect of AQ on Aβ_42_ aggregates, we incubated preformed Aβ_42_ fibrils with a 2.5 μM, 25 μM, and 250 μM concentrations of AQ (Figure 1B). Aβ_42_ was allowed to self-aggregate for at least 48 h to form mature fibrils (Figure S1A). The fluorescence intensities of Aβ_42_ fibrils treated with 2.5, 25, or 250 μM AQ were significantly reduced compared to those of the vehicle-treated Aβ_42_ fibrils from 60 to 96 h. In particular, the reductions in fluorescence intensities were more pronounced at 25 and 250 μM. The results showed that AQ partially inhibits the aggregation of Aβ and promotes the disaggregation of Aβ fibrils in a dose-dependent manner. Notably, AQ at a moderate concentration of 25 μM exhibited significant anti-aggregation and dis-aggregation activities compared to lower (2.5 μM) and higher (250 μM) concentrations.

### 3.2. Molecular Docking of AQ to Aβ

**Figure 2:**
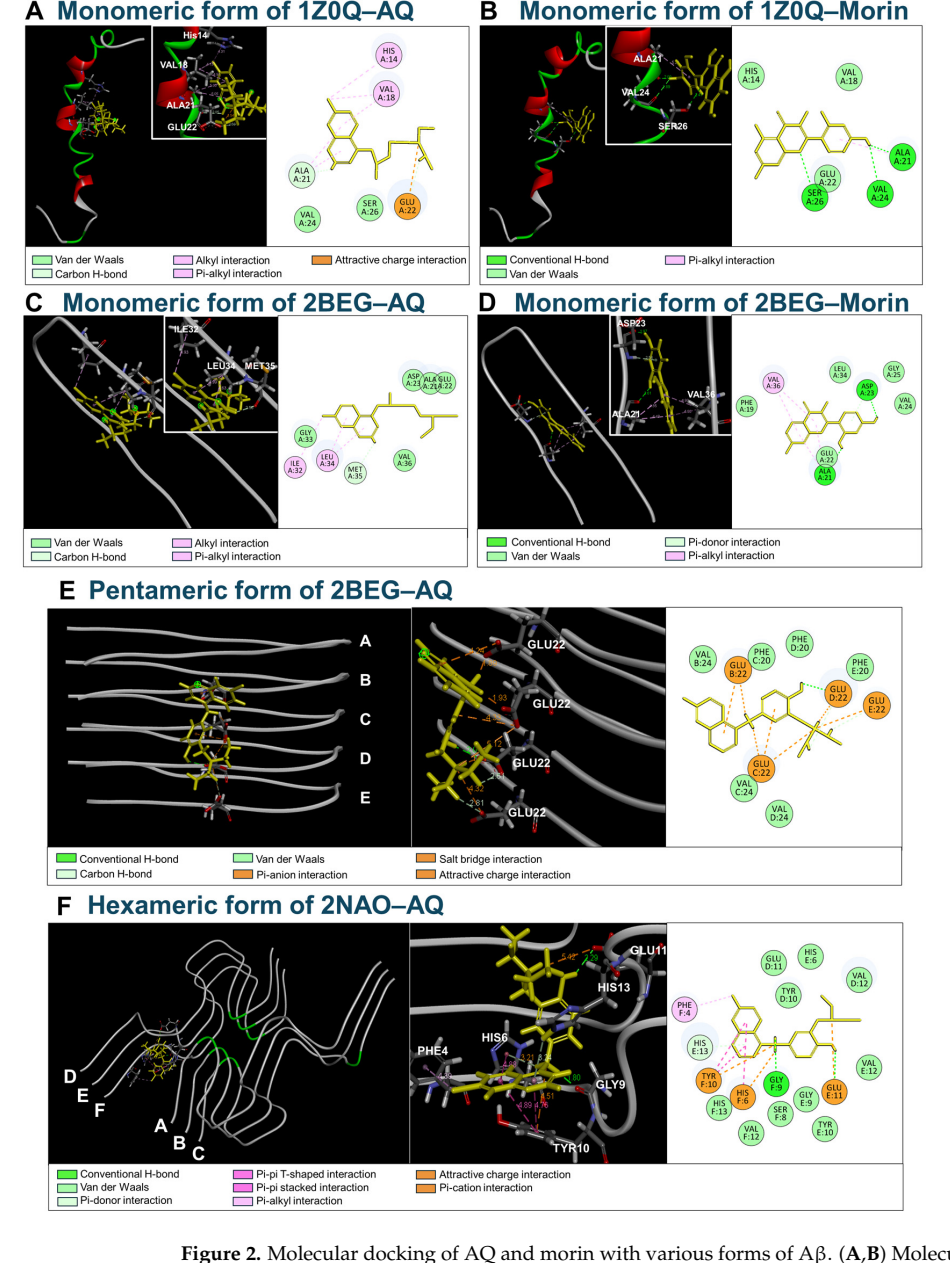
Molecular docking of AQ and morin with various forms of Aβ. (**A**,**B**) Molecular docking of the monomeric form of 1Z0Q with AQ and morin. (**C**,**D**) Molecular docking of the monomeric form of 2BEG with AQ and morin. (**E**) Molecular docking of the pentameric form of 2BEG with AQ. (**F**) Molecular docking of the hexameric form of 2NAO with AQ. The left panel shows ligands, such as AQ or morin (yellow), bound within the Aβ structure (gray). Aβ peptide chains are shown as gray ribbons, with green segments within the Aβ structure indicating regions adopting turn conformations during the docking. The middle panel provides an enlarged view of the binding pocket with key residues shown as sticks by the Aβ chain. The right panel shows 2D interaction maps, where each circle represents a residue involved in binding. In each circle, the top label indicates the residue name, while the bottom label shows the chain identifier followed by the residue number. The rectangular color blocks at the bottom indicate the types of interactions shown in the 2D diagram.

To investigate whether the anti-aggregation and disaggregation effects of AQ result from direct interactions with Aβ, we conducted molecular docking studies using various forms of Aβ. Aβ monomers are known to undergo a conformational change from an α-helical to a β-sheet structure depending on the composition of neuronal lipid membranes, surface charge, and hydrophobicity [22,23]. Since such an α-helix-to-β-sheet transition plays a critical role in the aggregation of Aβ, we performed molecular docking simulations to predict the binding of AQ and morin to Aβ monomers in both α-helical (1Z0Q) and β-sheet (2BEG) conformations. The −CDOCKER Energies for AQ and morin with the monomeric form of 1Z0Q were 4.0879 and 11.2416 kcal/mol, respectively. The Ligand Strain Energies of AQ and morin were calculated to be 21.7129 and 4.8304 kcal/mol, respectively (Table 2). In the complex with 1Z0Q, AQ formed hydrogen bonds with Ala21 and Glu22, hydrophobic interactions with Val18 (Alkyl) and His14, Val18 and Ala21 (π-alkyl). In contrast, morin established hydrogen bonds with Ala21, Val24 and Ser26 in the complex with 1Z0Q (Figure 2A,B). In addition, the −CDOCKER Energies for AQ and morin with the monomeric form of 2BEG were 7.2942 and 17.6258 kcal/mol, respectively, while their Ligand Strain Energies were determined to be 23.6421 and 4.9058 kcal/mol, respectively (Table 2). In the complex with 2BEG, AQ formed hydrogen bonds with Met35, hydrophobic interactions with Ile32 (Alkyl) and Leu34 (π-alkyl). However, morin showed hydrogen bonds with Ala21 and Asp23, as well as a π-donor H-bond with Asp23 and hydrophobic π-alkyl interaction with Ala21 and Val36 in the complex with 2BEG (Figure 2C,D). Interactions with monomeric forms of 1Z0Q and 2BEG are predicted to be stronger with morin compared to AQ. Next, to explore the interaction of AQ with Aβ aggregates, we investigated molecular docking studies using the pentameric form of 2BEG and the hexameric form of 2NAO. The −CDOCKER Energy of AQ was 45.1722 kcal/mol with the pentameric form of 2BEG and 23.3167 kcal/mol with the hexameric form of 2NAO, while the Ligand Strain Energies were 28.2477 kcal/mol and 23.8639 kcal/mol (Table 2). AQ formed hydrogen bonds with Glu22^D^, as well as a Carbon H-bond with Glu22^D^ and Glu22^E^ in the complex with pentameric form of 2BEG. AQ also established hydrogen bonds with Gly9 and Glu11^E^, as well as a π-donor H-bond with Glu22^D^ and Glu22^E^, hydrophobic interactions with Phe4^F^ (π-alkyl), His6^F^ (π-π T-shaped), and Try10^F^ and His6^F^ (π-π stacked) in the complex with hexameric form of 2NAO (Figure 2E,F). AQ exhibited relatively high Ligand Strain Energy when binding to Aβ aggregates containing stacked β-sheet structure compared to monomeric forms. Additionally, AQ showed stronger binding affinity and more abundant interactions with the aggregates compared to the monomeric forms, suggesting a preferential binding to the preformed aggregated form of Aβ. The relatively high ligand strain energy observed for AQ in the pentameric 2BEG complex suggests that the AQ requires a significantly high-energy conformation to bind, which may influence its binding affinity and kinetics.

### 3.3. Molecular Dynamics Simulation of AQ–Aβ Complex

**Figure 3:**
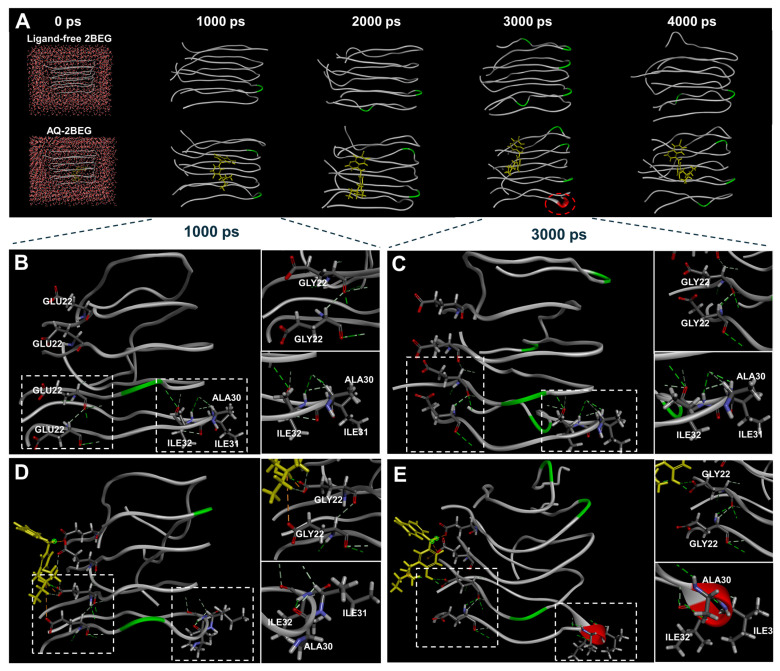
Molecular dynamics simulation of ligand-free 2BEG and the AQ–2BEG complex. (**A**) Snapshot of ligand-free 2BEG and AQ–2BEG complex over 4000 ps in a virtual environment. Representative images of ligand-free 2BEG at (**B**) 1000 ps and (**C**) 3000 ps. Representative images of the AQ–2BEG complex at (**D**) 1000 ps and (**E**) 3000 ps. In the figure, gray represents Aβ peptide chains, yellow indicates AQ, green segments within the Aβ chains highlight regions adopting turn conformations during the simulation, and red segments indicate regions of α-helix structures.

To predict the conformational changes in Aβ aggregates induced by the binding of AQ, we performed MD simulations. The total energies of ligand-free 2BEG and AQ–2BEG complex over 4000 ps were calculated to be −25.587 ± 0.003 and −27.200 ± 0.003 kcal/mol, respectively (Figure S5). Changes in the overall structure were analyzed through root mean square deviation (RMSD). The RMSD profiles of ligand-free 2BEG increased to 34.75 Å until approximately frame 120 and gradually decreased and stabilized within the range of 33.5–34.3 Å (Figure S4A). The RMSD profiles of the AQ–2BEG complex reached 34.50 Å by around frame 50 and subsequently stabilized within the same range of 33.5–34.3 Å (Figure S4B). These results suggest that the AQ–2BEG complex forms a stable structure more rapidly than ligand-free 2BEG. Additionally, we examined the structural images from 0 to 4000 ps to compare whether conformational changes between ligand-free 2BEG and the AQ–2BEG complex. Interestingly, at 3000 ps, an α-helix conformational change was observed in the AQ–2BEG complex that was not present in ligand-free 2BEG (Figure 3A). In the ligand-free 2BEG, the positions of the peptide chains remained unchanged, and no α-helix conformation was formed between 1000 and 3000 ps (Figure 3B,C). However, in the AQ–2BEG complex, several changes were observed. At 1000 ps, the 4th and 5th peptide chains of the AQ–2BEG complex showed three hydrogen bonds involving GLY22, along with one interaction with AQ (Figure 3D). At 3000 ps, only two hydrogen bonds remained at this site (Figure 3E). In addition, ALA30, ILE31, and ILE32 on the 5th peptide chain formed two hydrogen bonds at 1000 ps, which is three less than in ligand-free 2BEG. At 3000 ps, these residues formed hydrogen bonds at completely different positions compared to the ligand-free 2BEG. Notably, the newly formed hydrogen bonds may have contributed to the formation of a helical structure (Figure 3E). The MD simulations predict that AQ forms a stable complex with Aβ aggregates for a simulation time of 4000 ps and may contribute to disrupting the crossed β-sheet bonds by inducing an α-helix conformational change. Furthermore, we also performed MD simulations using the NPT ensemble, which showed a stable structure for the ligand-free 2BEG as well as the AQ–2BEG complex throughout the simulation time of 4000 ps (Figure S7).

### 3.4. Regulatory Effects of AQ on Tau K18

**Figure 4:**
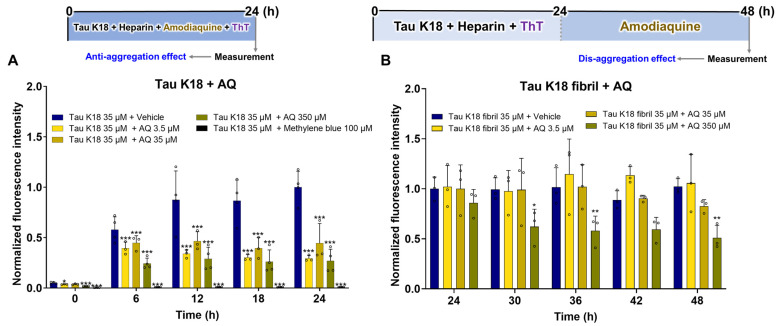
Regulatory effects of AQ on anti- and dis-aggregation of tau K18. ThT assay to assess the effect of AQ on the (**A**) aggregation of tau K18 and (**B**) dissociation of tau K18 fibrils. The vehicle group was treated with PBS. Methylene blue was used as a positive control for the tau aggregation inhibitor. Values are expressed as means ± standard deviations, n = 3–4 per group. Statistical significance was performed using two-way ANOVA, followed by Fisher’s LSD test. The F-statistics (F) are for (**A**) the row factor (F(4, 65) = 36.87, *p* < 0.0001) and column factor (F(4, 65) = 99.33, *p* < 0.0001), with a significant interaction detected (F(16, 65) = 7.220, *p* < 0.0001), and (**B**) the row factor (F(4, 40) = 0.7623, *p* = 0.5560) and column factor (F(3, 40) = 15.72, *p* < 0.0001), with a significant interaction also detected (F(12, 40) = 0.7012, *p* = 0.7407). * *p*-value < 0.05, ** *p*-value < 0.01, and *** *p*-value < 0.001 versus the vehicle-treated tau K18 group.

There is no reported evidence whether AQ inhibits the aggregation of tau or promotes the disassembly of preformed tau aggregates. Therefore, first we check the anti-aggregation effect of AQ on tau K18; we treated non-aggregated tau K18 with 3.5 μM, 35 μM, and 350 μM concentrations of AQ and methylene blue as a positive control (Figure 4A). The fluorescence intensities of tau K18 treated with 3.5 μM and 350 μM of AQ were markedly reduced fluorescence intensity at all times compared to vehicle-treated tau K18 (Figure 4A). Treatment with 35 μM of AQ resulted in a significant reduction in fluorescence intensity from 6 to 24 h compared to the vehicle group (Figure 4A). On the other hand, methylene blue consistently decreased fluorescence intensity compared to the vehicle group at all measured time points (Figure 4A). Next, to determine whether AQ can promote the dissociation of tau aggregates, we incubated tau fibrils with similar 3.5 μM, 35 μM, and 350 μM concentrations of AQ (Figure 4B). Tau K18 was aggregated for at least 24 h to form mature fibrils (Figure S3A). Treatment with 3.5 and 35 μM AQ exhibited no difference in fluorescence intensity compared to the vehicle group (Figure 4B). The fluorescence intensities of tau K18 fibrils treated with 350 μM of AQ showed a marked reduction starting at 30 h, exhibited a non-significant decreasing trend at 42 h, and became significantly reduced again at 48 h compared to those of the vehicle-treated fibrils (Figure 4B). Collectively, these findings indicate that AQ not only suppresses tau aggregation but also partially promotes the disassembly of tau preformed fibrils.

### 3.5. Molecular Docking of AQ to Tau

**Figure 5:**
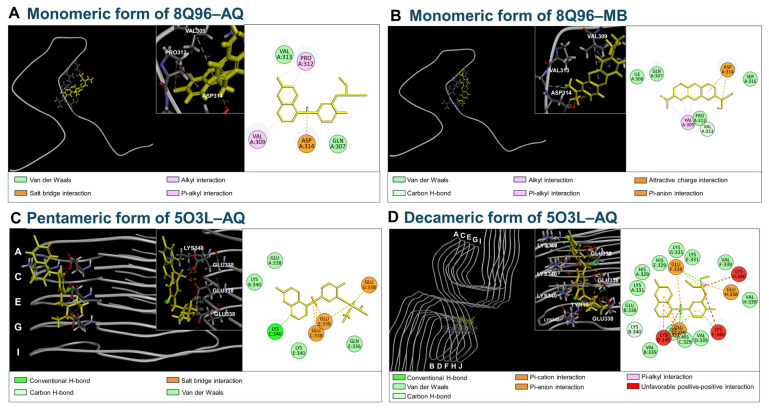
Molecular docking of AQ and methylene blue with various forms of tau. (**A**,**B**) Molecular docking of the monomeric form of 8Q96 with AQ and methylene blue. (**C**) Molecular docking of the pentameric form of 5O3L with AQ. (**D**) Molecular docking of the decameric form of 5O3L with AQ. The left panel shows a ligand, such as AQ or methylene blue (yellow), bound within tau structure (gray). Tau protein chains are shown as gray ribbons, with green segments within tau structure indicating regions adopting turn conformations during the docking. The middle panel provides an enlarged view of the binding pocket with key residues shown as sticks by tau chain. The right panel shows 2D interaction maps, where each circle represents a residue involved in binding. In each circle, the top label indicates the residue name, while the bottom label shows the chain identifier followed by the residue number. The rectangular color blocks at the bottom indicate the types of interactions shown in the 2D diagram.

To propose a possible mechanism underlying the modulatory effect of AQ on Tau aggregation, we performed in silico molecular docking studies. We performed docking simulations to predict the binding of AQ and methylene blue to tau monomers. The −CDOCKER Energies for AQ and methylene blue with the monomeric form of 8Q96 were 3.0534 and 7.7144 kcal/mol, respectively. The Ligand Strain Energies of AQ and methylene blue were calculated to be 23.2522 and 15.6787 kcal/mol, respectively (Table 3). AQ formed hydrophobic interactions with Pro312 (Alkyl), Pro312 and Val309 (π-alkyl) in the complex with 8Q96. In contrast, methylene blue established carbon hydrogen bond with Val313 and Asp314, hydrophobic interactions with Val309 (Alkyl and π-alkyl) in the complex with 8Q96 (Table 3, Figure 5A,B). Based on the interaction energies, methylene blue showed stronger interaction with monomeric forms of 8Q96 compared to AQ (Table 3). Next, to explore the interaction of AQ with tau aggregates, we investigated molecular docking studies using the pentameric form of 5O3L and the decameric form of 5O3L. The CDOCKER energy values for AQ were 27.9473 kcal/mol and 12.1302 kcal/mol for the pentameric and decameric forms of 5O3L, respectively. While the ligand strain energies were 29.4652 kcal/mol and 33.1491 kcal/mol for the pentameric and decameric forms of 5O3L, respectively (Table 3). In the complex with the pentameric form of 5O3L, AQ formed hydrogen bonds with Lys340^C^, as well as a carbon H-bond with Glu338^E^ and Glu338^G^. AQ also established hydrogen bonds with Glu338^D^ and Glu338^F^, as well as a carbon H-bond with Lys340^B^ and hydrophobic interaction with Lys340^D^ (π-alkyl) in complex with the decameric form of 5O3L (Figure 5C,D). AQ exhibited relatively high Ligand Strain Energy when binding to tau aggregates compared to Aβ form (Table 2 and Table 3). Molecular docking studies revealed that AQ forms direct interactions, including hydrogen and hydrophobic bonds, with 8Q96 and the monomeric and pentameric forms of 5O3L. Moreover, the relatively high ligand strain energy observed for AQ in the decameric 5O3L complex suggests that the AQ adopts a considerably strained conformation upon binding, which may influence the binding affinity and kinetics. These results suggest that the anti-aggregation and dissociation of preformed tau fibrils observed in the ThT assay may be mediated by direct interactions between AQ and tau.

### 3.6. Molecular Dynamics Simulation of AQ–Tau Complex

**Figure 6:**
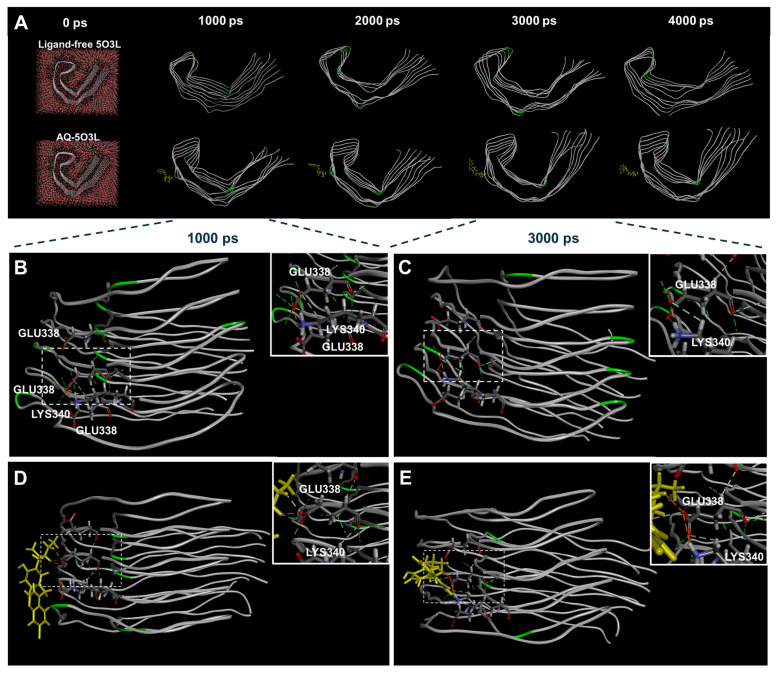
Molecular dynamics simulation of ligand-free 5O3L and AQ–5O3L complex. (**A**) Snapshot of ligand-free 5O3L and AQ–5O3L complex over 4000 ps in a virtual environment. Representative images of ligand-free 5O3L at (**B**) 1000 ps and (**C**) 3000 ps. Representative images of AQ–5O3L complex at (**D**) 1000 ps and (**E**) 3000 ps. In the figure, gray represents Aβ peptide chains, yellow indicates AQ, green segments within the Aβ chains highlight regions adopting turn conformations during the simulation.

To predict the conformational changes in tau aggregates induced by the binding of AQ to the mixed-polarity pocket, we performed MD simulations. The total energies of ligand-free 5O3L and AQ–5O3L complex over 4000 ps were calculated to be −70.640 ± 0.005 and −71.525 ± 0.005 kcal/mol, respectively (Figure S6). Changes in the overall structure were analyzed through root mean square deviation (RMSD). The RMSD pro-files of ligand-free 5O3L increased to 47 Å until approximately frame 40 and stabilized within the range of 47.0–48.0 Å (Figure S5A). The RMSD profiles of the AQ–5O3L complex reached 47 Å by around frame 40 and subsequently stabilized within the same range of 46.5–47.5 Å (Figure S5B). These results suggest that the structural stabilization of 5O3L occurs at a similar rate regardless of the presence or absence of AQ. Moreover, we examined the structural change from 0 to 4000 ps to compare whether conformational changes between ligand-free 5O3L and the AQ–5O3L complex (Figure 6A). However, both the ligand-free 5O3L and the AQ–5O3L complex showed no changes in the number or positions of hydrogen bonds at the region of interest between 1000 and 3000 ps (Figure 6B–E). Although molecular docking predicted that AQ binding would induce structural changes in tau aggregates, such conformational alterations were not observed in the MD simulations. Similarly, MD simulations using the NPT ensemble show no conformational changes in the ligand-free 5O3L and the AQ–5O3L complex (Figure S7). This lack of structural change may be attributed to the limited accessibility of AQ to its binding sites or to the intrinsic dynamic stability of the tau aggregates. Nevertheless, results from the ThT assay, in conjunction with the molecular docking analysis, support the potential of AQ to promote the disassembly and dissociation of tau aggregates.

### 3.7. Anti-Inflammatory Effect of AQ on Inflammatory Response Induced by Aβ_42_ and Tau K18

**Figure 7:**
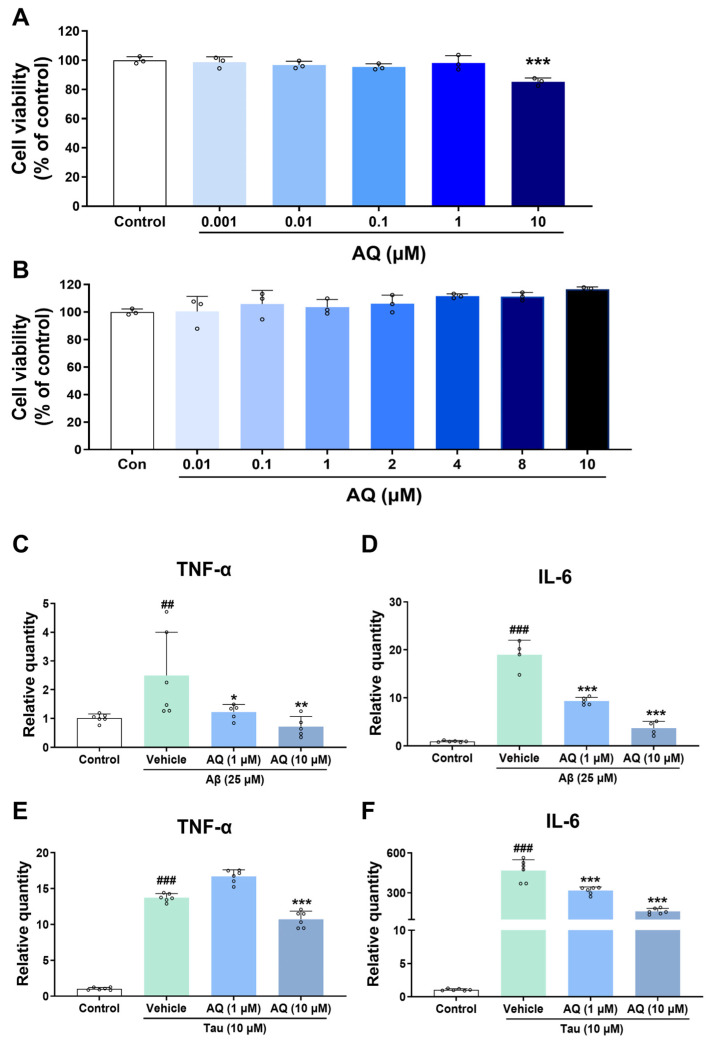
Anti-neuroinflammatory effect of AQ against inflammatory cytokines induced by Aβ and tau in BV2 microglia cells. (**A**) Cell viability of HT22 cells treated with several concentrations of AQ for 24 h. AQ showed no cytotoxic effects on HT22 cells at any concentration ranging from 0.001 to 1 μM, whereas 10 μM AQ significantly reduced cell viability. (**B**) Cell viability of BV2 cells treated with several concentrations of AQ for 24 h. AQ showed no cytotoxic effects on BV2 cells at any concentration ranging from 0.01 to 10 μM. (**C**,**D**) The mRNA levels of pro-inflammatory cytokines in BV2 cells 24 h after treatment with Aβ and AQ were measured using quantitative real-time PCR. (**E**,**F**) The mRNA levels of pro-inflammatory cytokines in BV2 cells 6 h after treatment with tau and AQ were measured using quantitative real-time PCR. Values are expressed as means ±  standard deviations. Statistical significance among the three groups was evaluated using one-way ANOVA followed by Fisher’s LSD test. The F-statistics (F) and t-values (t) are (**A**) F(5, 12) = 8.182, (**B**) F(7, 16) = 2.638, (**C**) F(3, 18) = 5.212 and t = 3.121 for treating Aβ_42_, t = 2.551 for treating Aβ_42_ with 1 μM AQ and t = 3.570 for treating Aβ_42_ with 10 μM AQ; (**D**) F(3, 15) = 118.5 and t = 17.97 for treating Aβ_42_, t = 9.273 for treating Aβ_42_ with 1 μM AQ and t = 13.93 for treating Aβ_42_ with 10 μM AQ; (**E**) F(3, 20) = 453.2 and t = 28.12 for treating tau and t = 6.650 for treating tau with 10 μM AQ; (**F**) F(3, 20) = 124.4 and t = 18.32 for treating tau, t = 5.917 for treating tau with 1 μM AQ and t = 12.04 for treating Aβ_42_ with 10 μM AQ. ^##^ *p*-value <  0.01 and ^###^ *p*-value <  0.001 versus control group; * *p*-value <  0.05, ** *p*-value <  0.001 and *** *p*-value <  0.001 versus vehicle group.

To determine whether AQ exhibits cellular cytotoxicity, we conducted a cell viability assay using BV2 microglial cells, which are known to produce pro-inflammatory cytokines in the brain with AD. Cell viability was assessed in HT22 neuronal cells treated with AQ at concentrations ranging from 0.001 to 10 μM, and reduced viability was observed at 10 μM (Figure 7A). Based on these results, we performed a WST-1 cell viability assay in BV2 cells to determine a concentration of AQ that does not induce cytotoxicity. In BV2 cells, cell viability was not affected at concentrations up to 10 μM (Figure 7B). Furthermore, to examine the inhibitory effect of AQ on the production of pro-inflammatory cytokines by microglia activated by Aβ and tau, we treated 1 and 10 μM of AQ to Aβ- or tau-treated BV2 microglial cells. Notably, Aβ and tau promote the expression of pro-inflammatory cytokines, such as TNF-α and IL-6, in microglia [24,25,26]. Thus, we measured the mRNA expression levels of TNF-α and IL-6 pro-inflammatory cytokines in microglial cells treated with Aβ and tau in the presence and absence of AQ. The level of TNF-α mRNA in Aβ-treated BV2 cells treated with vehicle was significantly increased compared to the control, whereas treatment with 10 μM of AQ markedly decreased the mRNA levels of TNF-α compared to the vehicle-treated group (Figure 7C). Likewise, IL-6 mRNA was strongly upregulated in Aβ-stimulated BV2 cells with vehicle treatment compared to the control. In contrast, the treatment of 1 and 10 μM AQ significantly reduced the expression of IL-6 mRNA compared to the vehicle group (Figure 7D). Vehicle-treated BV2 cells stimulated with tau showed enhanced the expression of both TNF-α and IL-6 compared to the control (Figure 7E,F). However, the treatment of 10 μM AQ in tau-treated BV2 cells significantly decreased the mRNA levels of TNF-α and IL-6 than those of vehicle-treated BV2 cells stimulated with tau (Figure 7E,F). These results indicate that AQ effectively suppresses the up-regulation of pro-inflammatory cytokines induced by Aβ and tau aggregates.

### 3.8. Inhibitory Effect of AQ on Production of Aβ

**Figure 8:**
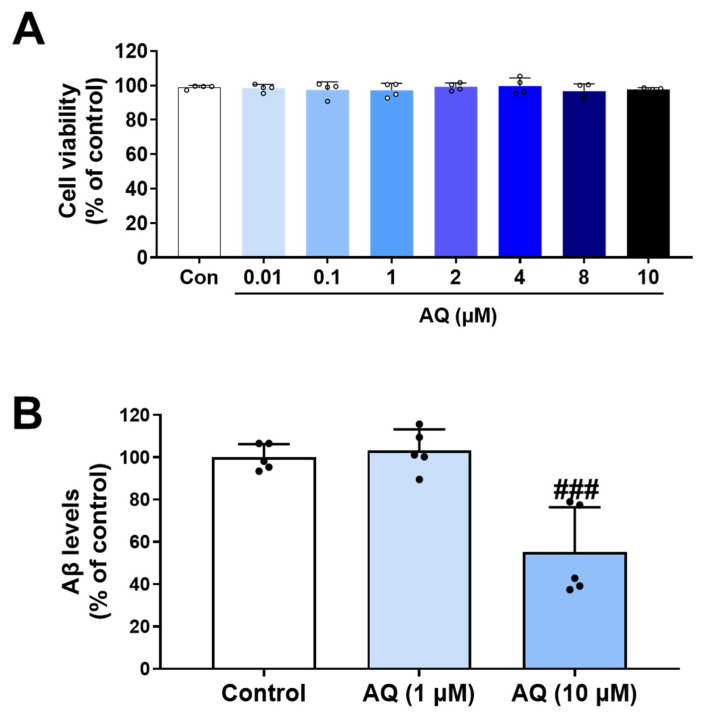
Inhibitory effect of AQ on Aβ production in APP-H4 cells. (**A**) Cell viability of APP-H4 cells treated with several concentrations of AQ for 24 h. AQ showed no cytotoxic effects on APP-H4 cells at any concentration ranging from 0.01 to 10 μM. (**B**) APP-H4 cells were treated with 1 and 10 μM AQ for 24 h and Aβ levels in conditioned media were quantified by ELISA. Values are expressed as means ± standard deviations. Statistical analysis was conducted by one-way ANOVA followed by Fisher’s LSD test. The F-statistics (F) and t-values (t) are (**A**) F(7, 24) = 0.3905, (**B**) F(2, 12) = 18.52 and t = 5.079 for 10 μM AQ versus control. ^###^ *p*-value < 0.001 versus control group.

The beneficial effects of AQ on diverse pathologies in AD have been reported in several studies [27]. According to a reported study, the 7-chloroquinolin-4-amine moiety of AQ was replaced with 2-aminomethylaniline and 2-aminomethylphenyl to synthesize two new compounds, **15a** and **16a** [27]. These compounds were found to inhibit the production of both Aβ_40_ and Aβ_42_ in SY5Y-APP695 human neuroblastoma cells. Based on our finding that AQ binds to Aβ and regulates its aggregation, a key feature of AD pathology, we further investigated the therapeutic effects of AQ on Aβ production, another feature of AD pathology. APP-H4 cells, a human neuroglioma line genetically modified to overexpress amyloid precursor protein (APP), are a valuable tool for AD research, such as studies on the mechanisms of Aβ production and processing, as well as the evaluation of therapeutic candidates [28]. Before examining Aβ production, we first assessed the cytotoxicity of AQ in APP-H4 cells. Cell viability was evaluated after treatment with AQ at concentrations of 0.01 to 10 μM, and no cytotoxicity was observed within this concentration range (Figure 8A). The effects of AQ on Aβ production were examined in APP-H4 cells using ELISA. Cells were treated with 1 μM or 10 μM AQ for 24 h, and Aβ levels in the conditioned media were quantified (Figure 8B). Interestingly, AQ treatment decreased the Aβ production in APP-H4 cells at 10 μM concentration. These findings together suggest that AQ is involved in regulating APP processing, potentially affecting Aβ production.

### 3.9. Prediction of Pharmacokinetics for AQ

PreADMET is a web-based tool that enables rapid and accurate prediction of the physical and pharmacokinetic properties of compounds in the early stages of drug development. Particularly, it is valuable for assessing the absorption, distribution, metabolism, and excretion (ADME) characteristics of potential drugs. By providing ADME predictions, PreADMET helps streamline the research process by identifying the pharmacological profiles of candidate compounds and facilitating the early elimination of compounds with a high likelihood of failure. In this context, we used PreADMET to evaluate the pharmacokinetic potential of AQ as a therapeutic agent and predict ADME properties (Table 4). AQ LogPo/w value was 4.95, indicating a strong affinity for lipids. The compound also demonstrated a high plasma protein binding (PPB) rate of 95.9%, which suggests that the concentration of free drug in plasma may be low, potentially extending the duration of its therapeutic effects. Moreover, the Caco-2 permeability was 41.78, and the human intestinal absorption (HIA) rate was 94.9%, indicating that AQ exhibits high cellular permeability and is suitable for oral administration. Additionally, AQ demonstrated a high blood–brain barrier (BBB) permeability value of 5.76, which emphasizes its strong capacity to penetrate the brain. Although AQ displayed favorable pharmacokinetic characteristics such as high intestinal absorption and BBB penetration, the very high PPB of AQ (95.9%) warrants careful consideration. A high PPB generally results in a reduced fraction of free drug in the circulation. This property implies that AQ may require a higher systemic dose to achieve therapeutic concentration in the CNS. The need for a higher dose of AQ could increase the risk of dose-dependent toxicities, which may complicate the clinical utility of AQ. Finally, in relation to metabolism, AQ was predicted to inhibit CYP2D6 but not to inhibit CYP2C19. The selective inhibition of CYP2D6 by AQ raises concerns about potential drug–drug interactions, especially when AQ is co-administered with CNS-active agents, such as antidepressants or antipsychotics, that are metabolized by CYP2D6. Furthermore, genetic polymorphisms in CYP2D6 could lead to substantial inter-individual variability in the pharmacological response and safety of AQ. On the other hand, the lack of CYP2C19 inhibition by AQ may reduce the risk of drug–drug interactions through the CYP2C19 pathway. AQ has a predicted total clearance rate of 1.265. It is also important to note that AQ, which is an FDA-approved antimalarial agent, has a known safety profile, including reports of hepatotoxicity as a notable adverse effect [29,30]. Therefore, while the ADME properties of AQ support its theoretical potential as a CNS therapeutic, the combination of high plasma protein binding, CYP2D6 inhibition, and a history of hepatotoxicity underscores the need for cautious dose optimization and safety evaluation of AQ before clinical translation.

## 4. Discussion

Herein, we demonstrate that AQ, a quinoline derivative, has the ability to modulate the aggregation and disassembly of Aβ and tau, two critical pathogenic factors of AD. Our ThT results showed that AQ significantly inhibited aggregation of Aβ at the 25 μM concentrations and promoted the dissociation of Aβ fibrils at all 2.5 μM, 25 μM, and 250 μM concentrations (Figure 1). We performed molecular docking and MD studies to elucidate a possible mechanism underlying the anti- and dis-aggregation effects of AQ on Aβ and its aggregates. The molecular docking results indicate that AQ was predicted to bind more strongly to the aggregated form of Aβ than to the monomeric form (Figure 2). In addition, molecular docking prediction suggested that AQ did not prefer to bind to hydrophobic sites critical for Aβ aggregation (Figure S6), suggesting that this could be mediated by a distinct mechanism. The MD results demonstrated that AQ may not interfere with the hydrophobic interactions of Aβ; instead, it has the potential to disrupt the crossed β-sheet bonds within Aβ aggregates (Figure 3). Next, ThT results showed that AQ effectively inhibited tau aggregation at all 3.5 μM, 35 μM, and 350 μM concentrations and promoted disassembly of tau aggregates at the 350 μM concentrations (Figure 4). Accordingly, we conducted molecular docking and molecular dynamics simulations to investigate the potential interaction and mechanisms underlying the anti-aggregation and disaggregation effects of AQ on tau and tau aggregates. The molecular docking results indicate that AQ binds favorably to both tau monomer and aggregated forms (Figure 5). However, MD simulations using both NVE and NPT ensembles did not reveal AQ-induced structural changes in tau aggregates up to 4000 ps simulation time (Figure 6 and Figure S7). This absence of structural changes may result from limited accessibility or the intrinsic dynamic stability of tau aggregates or may require a longer simulation time. In contrast, the ThT assay, which directly reflects protein–ligand interactions, indicated that AQ can still exert anti-aggregation and dissociation effects on tau. Finally, AQ, which modulates both the aggregation and disassembly of Aβ and tau, significantly suppressed the expression of pro-inflammatory cytokines that are upregulated by Aβ and tau in BV2 microglial cells (Figure 7). Similarly, AQ treatment inhibited the Aβ production levels in APP-H4 cells (Figure 8). Taken together, these findings suggest that AQ attenuates inflammatory activation and APP pathology by simultaneously regulating Aβ and tau aggregation.

AQ did not show a preference for binding to the hydrophobic site composed of residues critical for Aβ aggregation and fibril formation [9]. Instead, AQ interacted with both hydrophobic and hydrophilic residues within the mixed-polarity pocket (Figure S6). This binding mode can be explained by the amphipathic character of AQ. The quinoline core provides a hydrophobic aromatic surface that favors π-π stacking with nonpolar side chains, whereas the hydroxyl and tertiary amine substituents enable hydrogen bonding and electrostatic interactions with polar residues. Such dual functionality is reminiscent of other amphipathic inhibitors reported to interfere with Aβ aggregation, where the combination of aromatic moieties and polar or charged groups has been shown to modulate the conformational ensemble and reduce the formation of β-sheet structures [31,32]. AQ binding to the mixed-polarity pockets likely disrupts the inter-strand hydrogen bonding and aromatic stacking interactions that stabilize β-sheets. In the case of pre-formed fibrils, these hydrophobic and polar interactions may induce localized deformation by disrupting the delicate balance of weak forces, including hydrogen bonds, aromatic stacking, and hydrophobic contacts, that maintain fibril integrity. This disruption is expected to be most effective at fibril ends or structural defects, where bond-induced deformation could promote unraveling. This suggests that AQ functions not merely as an inhibitor, but as a modulator capable of influencing fibril structure depending on the aggregation state.

Quinoline derivatives have been widely reported to inhibit Aβ aggregation, and their therapeutic potential has been largely attributed to this inhibitory effect [11,12,14]. Nevertheless, growing evidence indicates that small molecules can exert concentration- or condition-dependent effects on Aβ aggregation. For instance, quinoline yellow has been shown to accelerate amyloid-like aggregation of myoglobin and α-lactalbumin by shortening the lag phase and promoting rapid transition to elongation [33,34]. Likewise, natural polyphenols such as epigallocatechin gallate (EGCG) and curcumin, although generally considered aggregation inhibitors, can paradoxically enhance fibrillation or increase aggregation rates under specific ratios or experimental conditions [35,36,37]. In particular, one study on EGCG showed that its effect depends strongly on the relative amount of EGCG to Aβ_42_ [35]. When the amount of EGCG was much higher than that of Aβ_42_, the aggregation rate accelerated; however, when the amount of EGCG was lower, aggregation was delayed. This result clearly shows that the same compound can have opposite effects on Aβ aggregation depending on its concentration and mixing ratio. These observations clearly illustrate that the same compound can exert opposing effects depending on concentration and stoichiometry, highlighting the non-linear and context-dependent nature of small molecule–amyloid interactions. In this regard, our study demonstrates that AQ exerts a concentration-dependent opposite effect on Aβ aggregation. At a low concentration (25 μM), AQ inhibited aggregation, whereas at a higher concentration (250 μM), it markedly accelerated the process, shortening the lag phase, promoting rapid elongation, and reaching a stable phase within 12 h (Figure 1A). Interestingly, the same concentration range of AQ was also capable of dissociating preformed fibrils, revealing its dual activity as both an aggregation accelerator and a fibril disruptor. These results suggest that AQ acts not simply as an inhibitor but as a modulator of Aβ dynamics that depends on the aggregation state. Several putative mechanisms may underlie these effects. First, AQ may bind to different structural states of Aβ. Binding to monomers or early oligomers could stabilize aggregation-prone conformations or bridge monomeric units, thereby accelerating nucleation. In contrast, interaction with mature fibrils may destabilize cross-β contacts and promote fragmentation, consistent with the observed disassembly of preformed fibrils. Second, AQ may differentially affect distinct kinetic phases of aggregation: it could enhance nucleation while destabilizing the structure of mature fibrils. Third, although potential assay artifacts, such as AQ–ThT interactions, were considered, our experiments excluded this possibility (Figures S1 and S3).

From a therapeutic perspective, these findings raise both opportunities and concerns. On one hand, the ability of AQ to disrupt preformed fibrils may be advantageous in facilitating the clearance of Aβ deposits. On the other hand, the concentration-dependent promotion of aggregation raises the concern that AQ might worsen amyloid pathology under certain conditions. However, previous studies with EGCG provide a different perspective. Some studies showed that EGCG can both redirect amyloidogenic polypeptides away from on-pathway fibril formation toward unstructured, off-pathway oligomers and remodel preformed fibrils of α-synuclein and Aβ into structurally distinct species with reduced neurotoxicity [36,38,39]. These results suggest that accelerated aggregation or remodeling does not necessarily increase toxicity and, in some cases, may represent a protective redirection of aggregation pathways. Consistent with this, our results show that AQ significantly reduced the mRNA levels of pro-inflammatory cytokines induced by Aβ and tau aggregates in BV2 microglial cells (Figure 7). This suggests that AQ may not simply promote pathogenic aggregation but rather modulate aggregate structures in a way that reduces the inflammatory response of aggregates.

Additionally, ThT assay results showed that AQ inhibits tau aggregation more effectively than Aβ, suggesting a stronger binding affinity for tau (Figure 1 and Figure 4). These effects may arise from the structural features of AQ. In the brain, tau accumulation is noninvasively detected using tau positron emission tomography (PET) radiolabeled probes, which are predominantly based on a quinoline scaffold [40,41]. In this regard, a previous study developed a quinoline-based fluorescent probe that selectively bound to tau aggregates over Aβ aggregates, exhibiting 3.5-fold higher selectivity for tau [42]. Interestingly, the developed probe shares several structural similarities with AQ, most notably the presence of a substituent at the 7-position of the quinoline ring. This structural similarity may allow AQ to bind more specifically to tau and inhibit its aggregation than Aβ.

Although MD simulations did not reveal significant conformational changes in the binding of AQ to tau aggregates, several possible mechanisms could explain the observed effects in the ThT assay. First, AQ may interfere with tau aggregation by binding to regions near nucleation or elongation sites. Our docking analyses demonstrated that AQ interacts with residues such as Lys340 and Glu338 within the β-sheet interface of tau fibrils (Figure 5C,D and Table 3). These interactions, even without global structural rearrangements, may sterically hinder the addition of monomers to the growing fibrils, thereby slowing down the kinetics of aggregation. Next, AQ binding could alter the local electrostatic or hydrophobic environment of tau aggregates. For instance, hydrogen bonding with Glu338 and hydrophobic interactions with Lys340 may shift the delicate balance of charge and polarity within the fibril surface. Such changes could destabilize intermolecular interactions essential for fibril propagation, even in the absence of large-scale structural distortions. Finally, AQ may stabilize intermediate or partially disassembled states of tau. By interacting with specific residues in the pentameric and decameric forms of tau, AQ might preferentially bind to transiently exposed pockets, thereby preventing the conversion of oligomeric intermediates into higher-order fibrils. This possibility is consistent with the relatively high ligand strain energy observed for AQ when binding on tau aggregates, suggesting that AQ adopts energetically demanding poses that could trap tau in less stable aggregation states. Taken together, these mechanisms suggest that AQ may modulate tau aggregation not only by direct conformational disruption but also by interfering with fibril growth dynamics, altering local interaction environments, and stabilizing aggregation intermediates.

AQ has demonstrated significant effects on AD pathology beyond modulation of Aβ and tau aggregation. In our previous study, treatment with AQ in 5XFAD mouse models led to a reduction in Aβ plaque accumulation, neuronal loss, and microgliosis [17]. Furthermore, impaired adult hippocampal neurogenesis was alleviated, and cognitive deficits were markedly improved. These findings suggest that AQ not only interferes with amyloid aggregation but also contributes to neuroprotection and functional recovery across multiple pathological features, including suppression of pro-inflammatory cytokines in AD. In addition, another study demonstrated that AQ treatment in adult rat hippocampal neural stem cells resulted in the upregulation of positive cell cycle regulators such as Cyclin D1, Cyclin A, and CDK2, along with increased expression of cell cycle progression markers including proliferating cell nuclear antigen (PCNA), minichromosome maintenance complex component 5 (MCM5), cell division cycle 25A (Cdc25a), and E2F1 [18]. This indicates that AQ may promote cell cycle progression by modulating key cell cycle-related molecules. This evidence supports the potential of AQ as a multi-target therapeutic candidate capable of addressing various aspects of AD pathology, including neuroinflammation, neurodegeneration, and impaired neurogenesis.

There are several limitations to this study. First, our ThT assays revealed that AQ exerts concentration-dependent dual effects on Aβ aggregation, inhibiting fibrillation at low concentrations but accelerating it at higher concentrations. Although our docking and MD simulation results support this mechanism by suggesting distinct binding preferences for monomeric versus aggregated Aβ, further studies are required to confirm these findings. In particular, ThT fluorescence assays alone cannot fully capture the structural complexity of Aβ species, and potential artifacts cannot be completely excluded. Therefore, future studies should include ThT-independent structural analyses, quantitative binding experiments such as isothermal titration calorimetry (ITC) or surface plasmon resonance (SPR), and time-course seeding assays to clarify the precise mechanism. Next, despite the lack of toxicity in BV2 and APP-H4 cells at 10 µM (Figure 7B and Figure 8A), this concentration was toxic to the HT22 neuronal cells (Figure 7A). This difference suggests that different cell lines may have distinct sensitivities to AQ. Given this finding, minimizing the potential neurotoxicity of AQ should be a key point in developing AQ-based therapeutic strategies for neurological diseases. One possible approach is to design drug delivery systems that selectively deliver AQ to target cells exhibiting inflammatory responses. For example, loading AQ onto nanoparticles conjugated with ligands that specifically recognize inflammation-related receptors could enable targeted delivery, thereby reducing systemic or neuronal toxicity. Therefore, future research on drug delivery strategies is needed to address this limitation.

## 5. Conclusions

Our study provides the first evidence that AQ, an antimalarial quinoline derivative, modulates both Aβ and tau pathologies, the two major factors of AD. AQ exhibited a dual regulatory effect on Aβ, partially inhibiting its aggregation at lower concentrations but promoting aggregation at higher concentrations, while consistently enhancing the disassembly of preformed fibrils. In addition, AQ significantly inhibited the aggregation of tau at all concentrations and promoted fibril disassembly at the highest concentration. Mechanistic insights from molecular docking and MD simulations further suggest that AQ may disrupt β-sheet structures in Aβ aggregates through preferential binding to mixed-polarity pockets, whereas its effects on tau through alternative binding mechanisms not fully captured by simulations. Moreover, AQ not only suppressed Aβ- and tau-induced pro-inflammatory cytokine expression in BV2 microglial cells but also reduced Aβ production in APP-H4 cells. Collectively, these findings highlight AQ as a promising multi-target therapeutic candidate that simultaneously regulates Aβ and tau aggregation, attenuates their associated inflammatory responses, and reduces Aβ pathology, thereby supporting its potential as a therapeutic candidate for AD.

## Figures and Tables

**Table 1:** PDB code for the macromolecule structure to simulate molecular docking.

Peptide/Protein	PDB Code	Simulation Models	Amino Acid Sequences
Aβ	1Z0Q	Monomeric form of 1Z0Q	^1^DAEFRHDSGYEVHHQKLVFFAEDVGSNKGAIIGLMVGGVVIA^42^
	2BEG	Monomeric form of 2BEG	^17^LVFFAEDVGSNKGAIIGLMVGGVVIA^42^
		Pentameric form of 2BEG	
	2NAO	Hexameric form of 2NAO	^1^DAEFRHDSGYEVHHQKLVFFAEDVGSNKGAIIGLMVGGVVIA^42^
Tau	8Q96	Monomeric form of 8Q96	^274^KVQIINKKLDLSNVQSKCGSKDNIKHVSGGGSCQIVYKPVDLSKVTSKCGSLGNIH^329^
	5O3L	Pentameric form of 5O3L	^306^VQIVYKPVDLSKVTSKCGSLGNIHHKPGGGQVEVKSEKLDFKDRVQSKIGSLDNITHVPGGGNKKIETHKLTF^378^
		Decameric form of 5O3L	

**Table 2:** Binding energies and interacting residues of AQ docked with multiple types of Aβ.

Ligands	Binding Energy			Interaction Residues		
	−CDOCKER Interaction Energy	Ligand Strain Energy ^#^	−CDOCKER Energy	H-Bond Interaction	Hydrophobic Interaction	Other
Monomeric form of 1Z0Q						
AQ	26.5208	21.7129	4.8079	Ala21 and Glu22 (Carbon H-bond)	Val18 (Alkyl), His14, Val18 and Ala21 (π-alkyl)	Glu22 (Attractive charge), Val24 and Ser26 (Van der Waals)
Morin	16.0720	4.8304	11.2416	Ala21, Val24 and Ser26 (H-bond)	Ala21 (π-alkyl)	His14, Val18 and Glu22 (Van der Waals)
Monomeric form of 2BEG						
AQ	30.9364	23.6421	7.2942	Met35 (Carbon H-bond)	Ile32 (Alkyl), Leu34 (π-alkyl)	Asp23, Ala21, Glu22, Gly33 and Val36 (Van der Waals)
Morin	22.5316	4.9058	17.6258	Ala21 (H-bond), Asp23 (H-bond and π-donor H bond)	Ala21 and Val36 (π-alkyl)	Phe19, Glu22, Val24, Gly25 and Leu34 (Van der Waals)
Pentameric form of 2BEG						
AQ	73.4199	28.2477	45.1722	Glu22^D^ (H-bond and Carbon H-bond), Glu22^E^ (Carbon H-bond)	-	Glu22^BCD^ (Salt bridge), Glu22^CE^ (Attractive charge), Glu22^BC^ (π -anion), Phe20^CDE^ and Val24^BCD^ (Van der Waals)
Hexameric form of 2NAO						
AQ	47.1806	23.8639	23.3167	Gly9^F^ and Glu11^E^ (H-bond), His13^E^ (π-donor H bond)	Phe4^F^ (π-alkyl), His6^F^ (π-π T-shaped), Try10^F^ and His6^F^ (π-π stacked)	Glu11^E^ (Attractive charge), Tyr10^F^ and His6 (π-cation), His6^E^, SER8^F^, GLY9^E^, Glu11^D^, Val12^DEF^, Try10^ED^ and His13^F^ (Van der Waals)

^B–F^ These letters indicate the individual chains that make up the layered Aβ fibril structure. ^#^ Ligand Strain Energy refers to the energy required to deform the ligand from its optimal conformation to the bound state. $Ligand\;Strain\;Energy=\left|\left(-CDOCKER\;Interaction\;Energy\right)\;-\;\left(-CDOCKER\;Energy\right)\right|$.

**Table 3:** Binding energies and interacting residues of AQ docked with multiple types of tau.

Ligands	Binding Energy			Interaction Residues		
	−CDOCKER Interaction Energy	Ligand Strain Energy ^#^	−CDOCKER Energy	H-Bond Interaction	Hydrophobic Interaction	Other
Monomeric form of 8Q96						
AQ	26.3056	23.2522	3.0534	-	Pro312 (Alkyl), Pro312 and Val309 (π-alkyl)	Val313 and Gln307 (Van der Waals)
Methylene blue	23.3931	15.6787	7.7144	Val313 and Asp314 (Carbon H-bond)	Val309 (Alkyl and π-alkyl)	Asp314 (Attractive charge and π-anion), Ile308, Gln307 and Ser316 (Van der Waals)
Pentameric form of 5O3L						
AQ	57.4125	29.4652	27.9473	Lys340^C^ (H-bond), Glu338^EG^ (Carbon H-bond)	-	Glu338^A^, Lys340^AE^ and Gln336^E^ (Van der Waals), Glu338^CEG^ (Salt bridge)
Decameric form of 5O3L						
AQ	45.2739	33.1491	12.1302	Glu338^DF^ (H-bond), Lys340^B^ (Carbon H-bond)	Lys340^D^ (π-alkyl)	His329^ACE^, Lys331^ACEG^, Glu338^B^ and Val^BDFH^ (Van der Waals), Glu338^FH^ (Salt bridge), Glu338^DFH^ (Attractive charge), Lys340^DF^ (π-cation), Glu338^D^ (π-anion)

^A–H^ These letters indicate the individual chains that make up the layered tau fibril structure. ^#^ Ligand Strain Energy refers to the energy required to deform the ligand from its optimal conformation to the bound state. $Ligand\;Strain\;Energy=\left|\left(-CDOCKER\;Interaction\;Energy\right)\;-\;\left(-CDOCKER\;Energy\right)\right|$.

**Table 4:** Prediction of pharmacokinetics for AQ.

Pharmacokinetic Properties								
Compound name	logPo/w ^a^	PPB ^b^	Caco2 ^c^	HIA ^d^	BBB ^e^	CYP _2C19 _inhibition ^f^	CYP 2D6 _inhibition ^g^	Total Clearance ^h^
AQ	4.95	95.9	41.78	94.9	5.76	Non	Inhibitor	1.265

^a^ Partition coefficient between n-octanol and water (LogPo/w > 1, lipophilicity; logPo/w < 1, hydrophilicity). ^b^ Plasma protein binding (PPB < 90%, weakly binds to proteins in plasma.; PPB > 90%, strongly binds to proteins in plasma). ^c^ Caco2 cell permeability. ^d^ Human intestinal absorption (HIA ≤ 20%, poor absorption into the intestines; 20% < HIA ≤ 70%, moderate absorption into the intestines; 70% < HIA ≤ 100%, well absorption into the intestines). ^e^ Penetration into the brain (<0.1, low penetration into the brain; 0.1–2.0, moderate penetration into the brain). ^f^ Cytochrome P450 2C19 inhibition. ^g^ Cytochrome P450 2D6 inhibition. ^h^ Total clearance (log mL/min/kg).

## Data Availability

The original contributions presented in this study are included in the article and Supplementary Materials. Further inquiries can be directed to the corresponding authors.

## Supplementary Materials

The following supporting information can be downloaded at: https://www.mdpi.com/article/10.3390/pharmaceutics17111417/s1. Figure S1: Fluorescence intensity of Aβ_42_ and spectral interference analysis with thioflavin T (ThT). Figure S2: Purification of recombinant tau K18. Figure S3: Fluorescence intensity of tau K18 and spectral interference analysis with Thioflavin T (ThT). Figure S4: Total energy and root mean square deviation (RMSD) profiles of pentameric form of 2BEG. Figure S5: Total energy and root mean square deviation (RMSD) profiles of pentameric form of 5O3L. Figure S6: Binding of AQ with mixed-polarity and hydrophobic pockets in the pentameric form of 2BEG. Figure S7: Molecular dynamics simulation of the ligand-free 2BEG, AQ–2BEG complex, ligand-free 5O3L, and AQ–5O3L complex.

## Abbreviations

The following abbreviations are used in this manuscript:

Aβ	Amyloid-β
AD	Alzheimer’s disease
ADME	Absorption, distribution, metabolism, and excretion
APP	Amyloid precursor protein
AQ	Amodiaquine
BBB	Blood–brain barrier
Cdc25a	Cell division cycle 25A
CDK2	Cyclin-dependent kinase 2
CHARMm	Chemistry at HARvard Macromolecular Mechanics
Cq	Quantification cycle
DPBS	Dulbecco’s phosphate-buffered saline
E2F1	E2F transcription factor 1
EGCG	Epigallocatechin gallate
ELISA	Enzyme-linked immunosorbent assay
GAPDH	Glyceraldehyde-3-phosphate dehydrogenase
HIA	Human intestinal absorption
IL-6	Interleukin-6
ITC	Isothermal titration calorimetry
LSD	Least significant difference
MB	Methylene blue
MCM5	Minichromosome maintenance complex component 5
MD	Molecular dynamics
NFTs	Neurofibrillary tangles
NR	Newton-Raphson
PCNA	Proliferating cell nuclear antigen
PET	Positron emission tomography
PHFs	Paired helical filaments
PPB	Plasma protein binding
PS	Picosecond
RMS	Root mean square
RMSD	Root mean square deviation
SD	Standard deviations
SPR	Surface plasmon resonance
ThT	Thioflavin T
TNF-α	Tumor necrosis factor-alpha
WST-1	Water-soluble tetrazolium-1
