# Supplementary material for: Amodiaquine Modulates Aggregation and Disassembly of Amyloid-β and Tau and Attenuates Neuroinflammatory Responses and Aβ Production
Source: Pharmaceutics. 2025 Oct 31;17(11):1417. doi: 10.3390/pharmaceutics17111417 (PMC12655649; doi:10.3390/pharmaceutics17111417)
Supplement: Supplementary file 1 [file pharmaceutics-17-01417-s001.zip › pharmaceutics-3922587-supplementary.pdf]

pharmaceutics

MDPI

Supplementary Materials: Amodiaquine Modulates Aggregation and Disassembly of Amyloid-β and Tau and Attenuates Neuroinflammatory Responses and Aβ Production

Sinae Jang, Sujin Kim, Na-Hyun Kim, Soo Jung Shin, Vijay Kumar, Jeong Gyu Son, Minseok Lee, Choon-gil Kim, Eun-Kyung Lim, Hyunju Chung, Young Ho Koh, Yunkwon Nam and Minho Moon

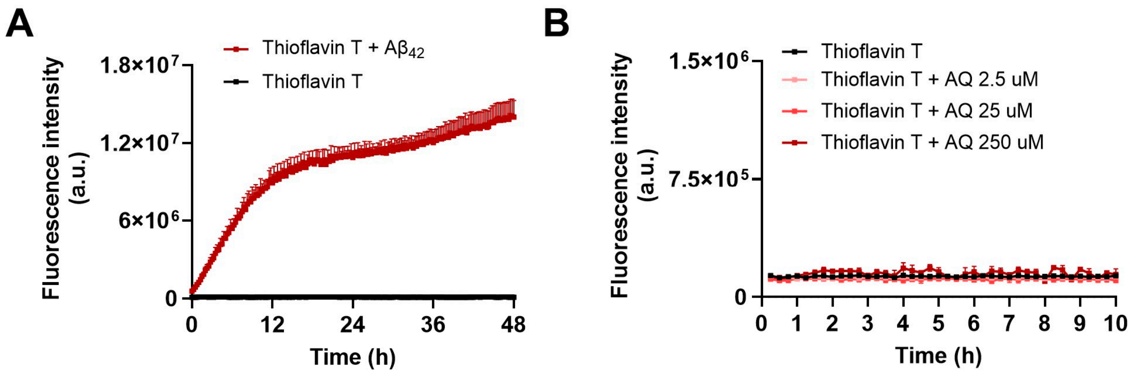

Figure S1. Fluorescence intensity of $A\beta_{42}$ and spectral interference analysis with thioflavin T (ThT). (**A**) Fluorescence intensity measurement of 25 μM $A\beta_{42}$ using a ThT assay. (**B**) Analysis of fluorescence spectra interference between ThT and 2.5, 25, and 250 μM of AQ. Values are expressed as means ± standard deviations.

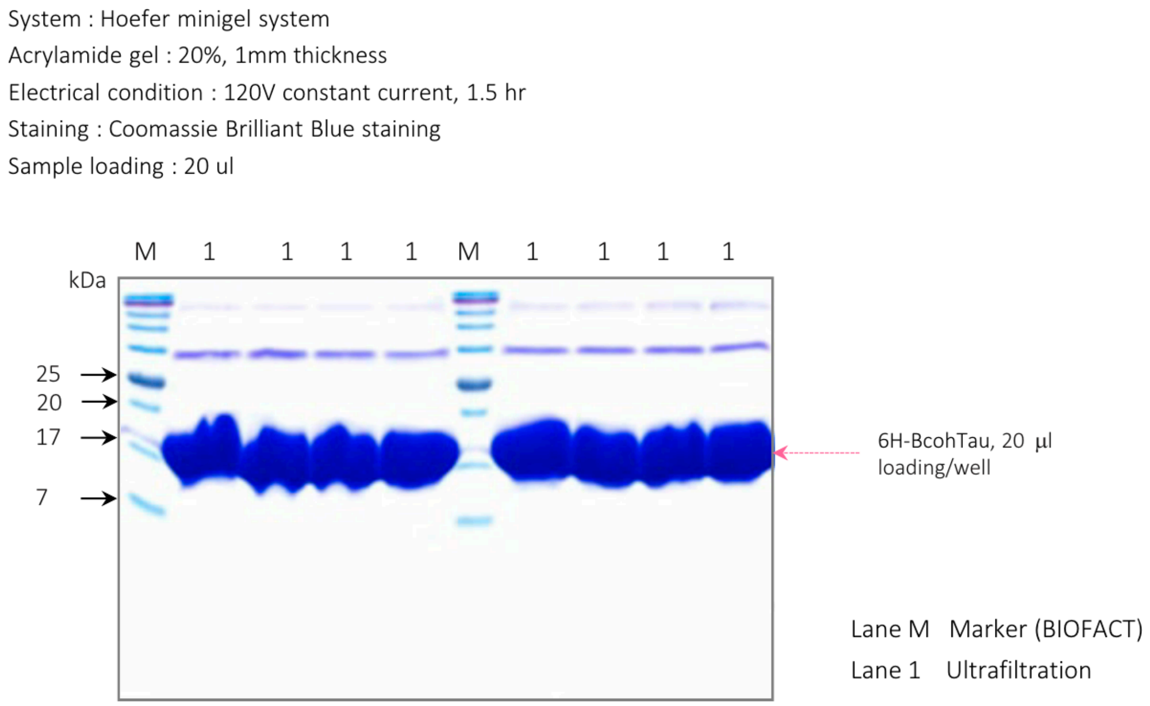

Figure S2. Purification of recombinant tau K18. Electrophoresis of sodium dodecyl sulfate-polyacrylamide gels confirmed the presence of recombinant tau K18. The molecular weight of the protein is indicated by the protein marker (kDa) and the red arrow indicates the purified protein.

 https://doi.org/10.3390/pharmaceutics17111417

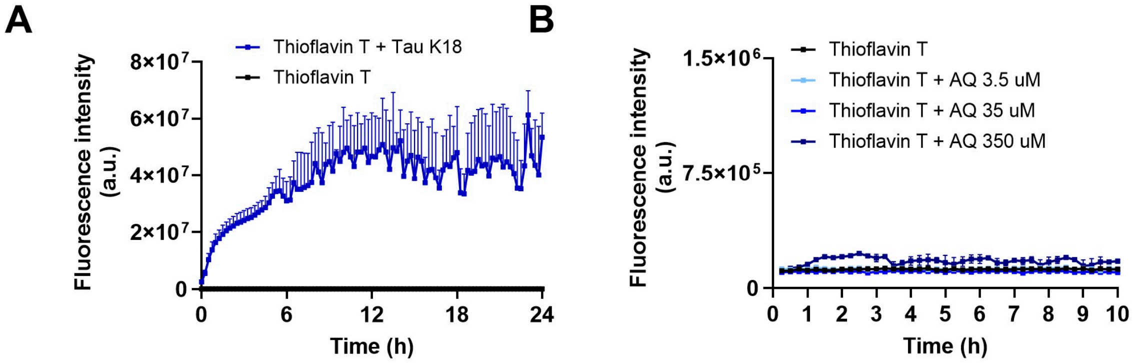

Figure S3. Fluorescence intensity of tau K18 and spectral interference analysis with Thioflavin T (ThT). (**A**) Fluorescence intensity measurement of tau K18 using ThT assay. (**B**) Analysis of fluorescence spectra interference between ThT and 3.5, 35, and 350 μM of AQ. Values are expressed as means ± standard deviations.

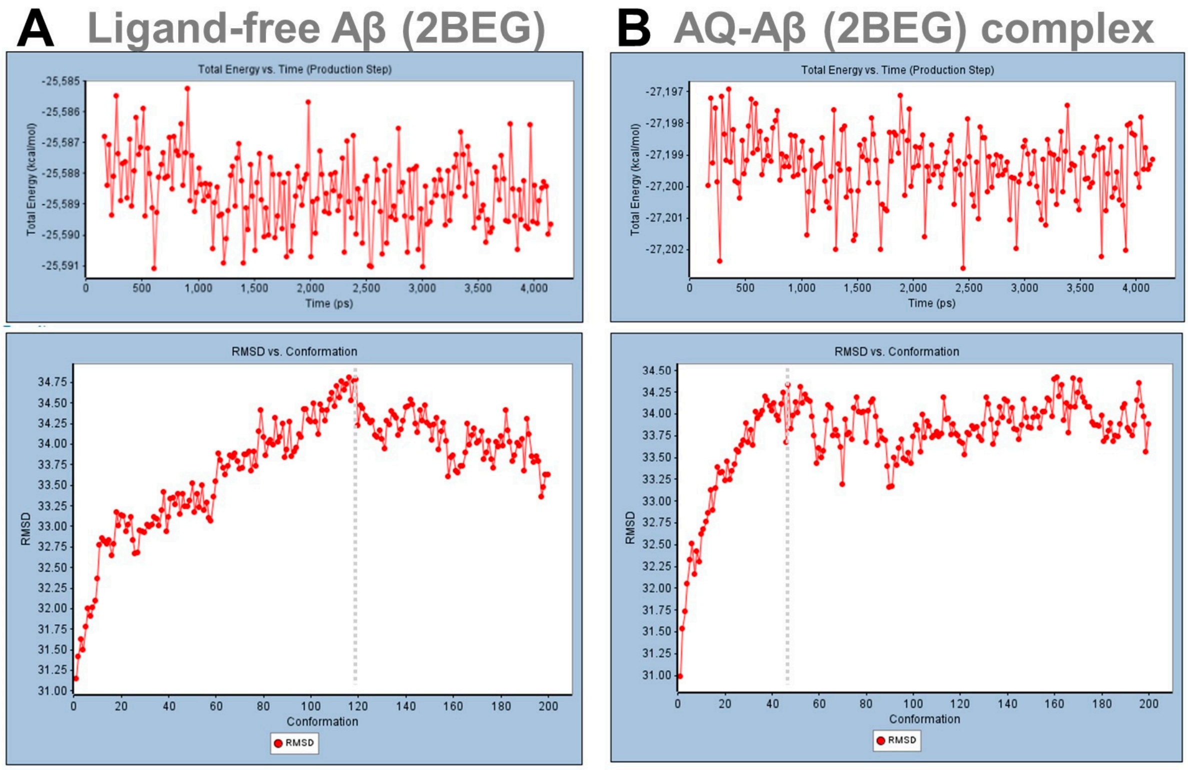

Figure S4. Total energy and root mean square deviation (RMSD) profiles of pentameric form of 2BEG (**A**) without AQ and (**B**) with AQ. Molecular dynamics (MD) simulations were performed for 4000 ps, storing 200 conformations. The RMSD value represents the average distance between the atomic positions of each conformation obtained from MD simulation (200 structures in total) and the initial structure at the start of the simulation, with 2BEG used as the reference. A higher RMSD indicates greater structural deviation, and a plateau in the graph shows a stabilized structure.

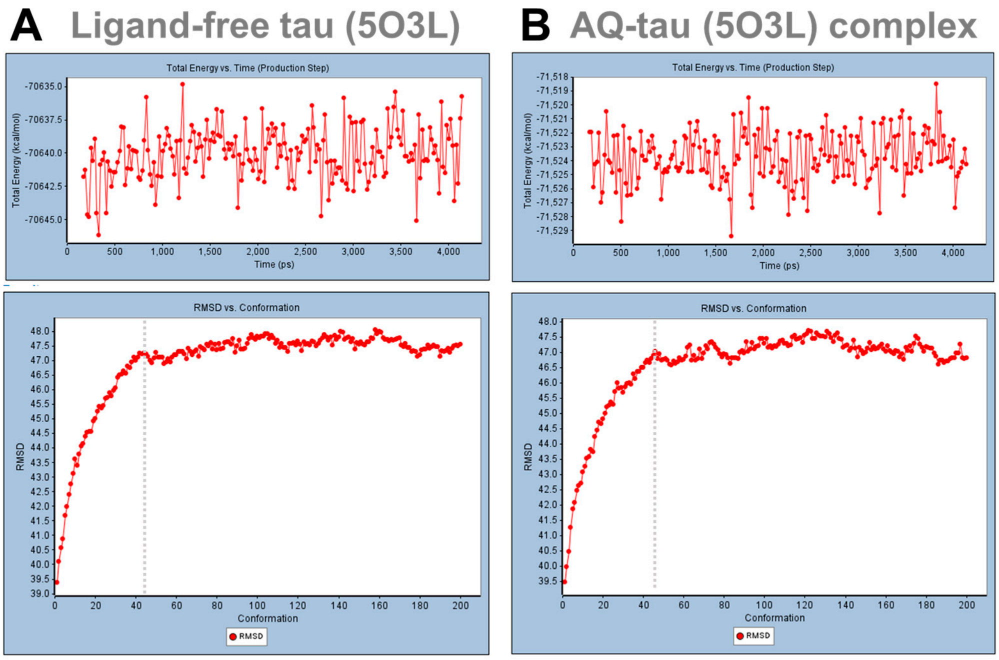

Figure S5. Total energy and root mean square deviation (RMSD) profiles of pentameric form of 5O3L (**A**) without AQ and (**B**) with AQ. Molecular dynamics (MD) simulations were performed for 4000 ps, storing 200 conformations. The RMSD value represents the average distance between the atomic positions of each conformation obtained from MD simulation (200 structures in total) and the initial structure at the start of the simulation, with 5O3L used as the reference. A higher RMSD indicates greater structural deviation, and a plateau in the graph shows a stabilized structure.

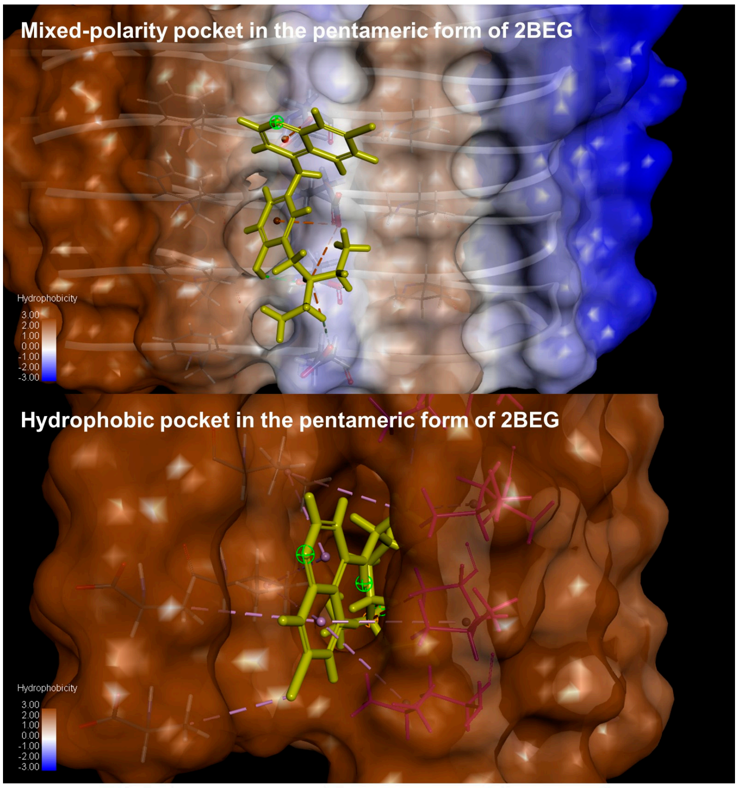

Ligand	-CDOCKER Interaction Energy	Ligand Strain Energy	-CDOCKER Energy
Mixed-polarity pocket in the pentameric form of 2BEG			
AQ	73.4199	28.2477	45.1722
Hydrophobic pocket in the pentameric form of 2BEG			
AQ	32.0709	30.1955	1.8754

Figure S6. Binding of AQ with mixed-polarity and hydrophobic pockets in the pentameric form of 2BEG. Representation of AQ bound within a mixed-polarity pocket of the pentameric 2BEG (Upper panel). Representation of AQ bound within a hydrophobic pocket of the pentameric 2BEG (Lower panel). The −CDOCKER Energy showed that AQ binds more strongly in mixed-polarity pockets than in hydrophobic pockets. The surface is colored according to hydrophobicity, where brown indicates hydrophobic regions and blue indicates hydrophilic regions.

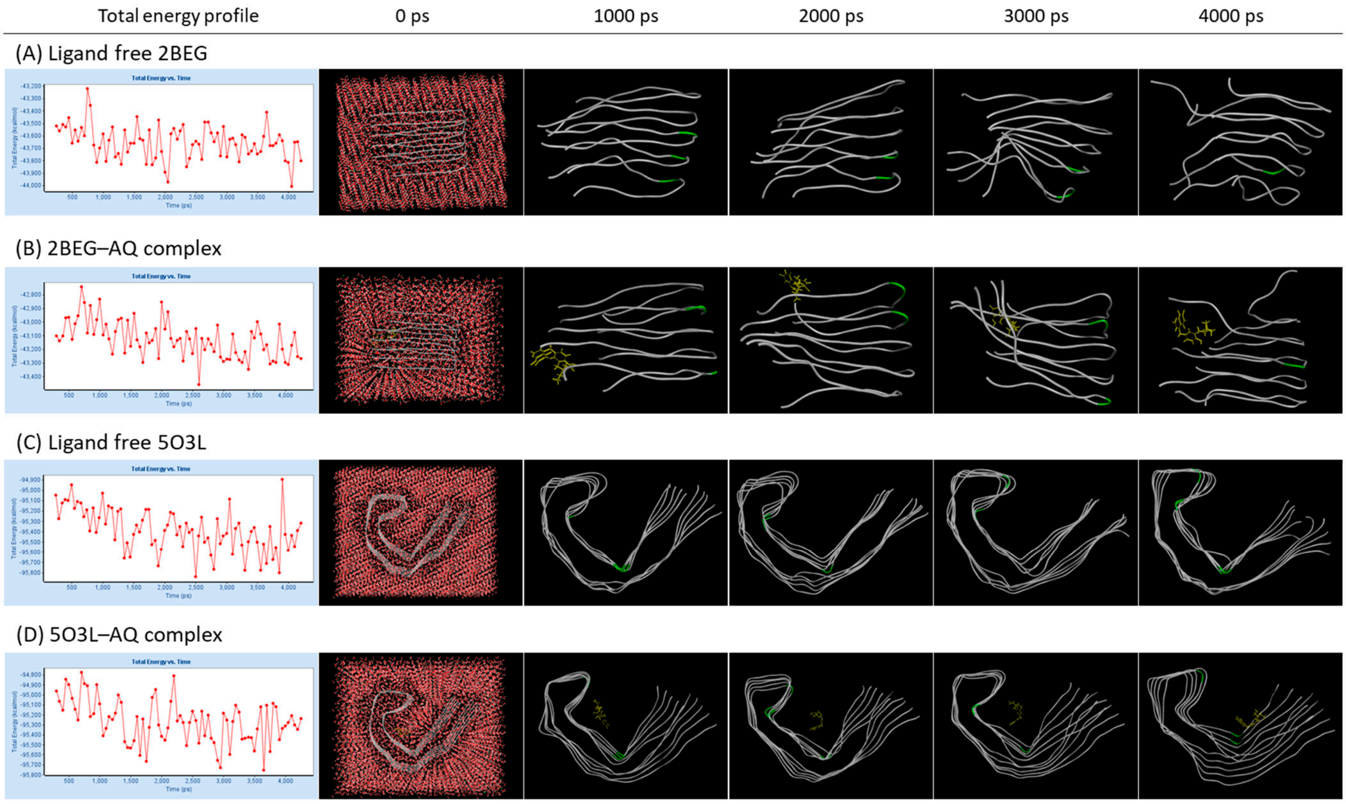

Figure S7. Molecular dynamics simulation of the ligand-free 2BEG, AQ–2BEG complex, ligand-free 5O3L, and AQ–5O3L complex. (A) Snapshot of ligand-free 2BEG for total energy profile (first panel) and representative images of 0 ps, 1000 ps, 2000 ps, 3000 ps, and 4000 ps in a virtual environment. (**B**) Snapshot of 2BEG–AQ complex for total energy profile (first panel) and representative images of 0 ps, 1000 ps, 2000 ps, 3000 ps, and 4000 ps in a virtual environment. (**C**) Snapshot of ligand-free 5O3L for total energy profile (first panel) and representative images of 0 ps, 1000 ps, 2000 ps, 3000 ps, and 4000 ps in a virtual environment. (**D**) Snapshot of 5O3L–AQ complex for total energy profile (first panel) and representative images of 0 ps, 1000 ps, 2000 ps, 3000 ps, and 4000 ps in a virtual environment.
